# Estimating the risk of acute kidney injury associated with use of diuretics and renin angiotensin aldosterone system inhibitors: A population based cohort study using the clinical practice research datalink

**DOI:** 10.1186/s12882-019-1633-2

**Published:** 2019-12-30

**Authors:** Jemima Scott, Tim Jones, Maria Theresa Redaniel, Margaret T. May, Yoav Ben-Shlomo, Fergus Caskey

**Affiliations:** 1 Richard Bright Renal Unit, Southmead Hospital, Bristol, BS10 5NB UK; 2 Bristol Medical School: Population Health Sciences, University of Bristol, Bristol, UK; 3 NIHR CLAHRC West, University of Bristol, Bristol, UK; 4 UK Renal Registry, Bristol, UK

**Keywords:** Acute kidney injury, Diuretics, Renin-angiotension-aldosterone inhibitors

## Abstract

**Background:**

The risk of acute kidney injury (AKI) attributable to renin angiotensin aldosterone (RAAS) inhibitors and diuretics remains unclear.

**Methods:**

We conducted a prospective cohort study using the Clinical Practice Research Datalink (2008–2015) linked to Hospital Episode Statistics – Admitted Patient Care and Office for National Statistics mortality data. Patients were included if they had one or more chronic diagnoses requiring medication. Exposed patients had a first ever prescription for RAAS inhibitors/diuretics during the study period. AKI risk associated with exposure was determined by multivariable Cox regression, propensity score-adjusted Cox regression and a prior event rate ratio (PERR) analysis.

**Results:**

One hundred forty thousand nine hundred fifty-two individuals were included. Increased AKI risk in the exposed group was demonstrated in both the multivariable and propensity score-adjusted cox regressions (HR 1.23 (95% CI 1.04–1.45) and HR 1.24 (1.05–1.47) respectively). The PERR analysis provided a similar overall hazard ratio with a wider confidence interval (HR 1.29 (0.94–1.63)). The increased AKI risk in the exposed group was present only in those receiving two or more antihypertensives. Absolute AKI risk was small.

**Conclusions:**

RAAS inhibitors/diuretics result in an increased risk of AKI. The absolute increase in AKI risk is small, however, and needs to be considered in the context of any potential benefits.

## Background

The reported incidence of acute kidney injury (AKI) in community-dwelling adults and hospital inpatients varies significantly depending on the criteria used [1]. A recent meta-analysis concluded that worldwide, one in five adults and one in three children experience an episode of AKI during an inpatient admission [2]. Studies in high-income countries have reported an incidence of AKI of 522/100,000 people per year in the community, [3] and up to 22.7/100 in an inpatient setting [4]. The incidence of AKI is likely to be increasing [3, 5] due to an ageing population with increased comorbidity and polypharmacy.

There is significant morbidity, mortality and economic cost associated with AKI. A meta-analysis of adverse outcomes following AKI conducted in 2009 [6] found the risks of chronic kidney disease (CKD) and end-stage kidney disease (ESKD) following a single episode of AKI to be 7.8 and 4.9/100 patient-years, respectively. Even mild AKI (a rise in serum creatinine of less than or equal to 25%) was associated with a 70% increase in mortality. In 2014 the financial burden associated with AKI in the United Kingdom (UK) was estimated to be £1.02 billion, just over 1% of the annual National Health Service budget [7].

AKI may result from reduced kidney perfusion, intrinsic renal disease or obstructive causes, with the first of these accounting for 75% of AKI episodes in hospital settings [8]. Risk factors include increasing age, sepsis, hypotension and chronic conditions (diabetes mellitus, congestive cardiac failure (CCF), CKD, atherosclerotic peripheral vascular disease, liver disease) [9]. Certain medications, including non-steroidal anti-inflammatories (NSAIDs), diuretics and agents that inhibit the renin-angiotensin-aldosterone (RAAS) axis have also been suggested to increase the risk of AKI in epidemiological studies, [10–12] however the absolute risk of AKI amongst these individuals is unknown.

The absolute risk of AKI monsgt maintenance users of RAAS inhibitors and diuretics is unknown. This study aims to determine the absolute and relative risk of AKI in maintenance users of RAAS inhibitors and diuretics in a “real-world” setting of community-dwelling comorbid adults.

## Methods

### Data source and population

We conducted a prospective cohort study using electronic medical records from the Clinical Practice Research Datalink (CPRD) GOLD. At the time of data extraction (July 2016), CPRD included records from 701 general practices in the UK, and over 16 million patients [13]. The demographics of registered patients are representative of the UK [14]. CPRD data have been validated, audited, and quality checked [15]. Primary care data from CPRD GOLD were linked to the Hospital Episode Statistics – Admitted Patient Care (HES-APC) database, Office for National Statistics Mortality data, and Indices of Multiple Deprivation, as long as patients were eligible for linkage (around 60% of CPRD patients). The study protocol was approved by the Independent Scientific Advisory Committee (ISAC) for MHRA Database Research (protocol number: 16_030R).

### Code lists

We defined our chronic conditions, outcome, exposure, and covariables using medical and product codes within CPRD, as well as values of specific blood test results. Medical codes relate directly to clinical Read codes, whilst product codes relate to the British National Formulary. Medications for chronic conditions were cross-checked against European guidelines on classification of medicines, [16] and by a clinical expert (F.C.). Additionally, we used the International Classification of Diseases (version 10) (ICD-10) to identify diagnosis of AKI in HES-APC. Code lists are available from [https://github.com/jonestim2002/aki_raas_diuretics].

### Patients

We included patients from CPRD with indications of any of five chronic conditions: CKD, diabetes, CCF, hypertension, or ischaemic heart disease. Patients had to have a valid month and year of birth, sex, and registration date. We required a reasonable ordering of events for inclusion: diagnosis of first chronic condition after the practice up-to-standard date (representing reasonable quality of data collection from that practice); exposure date after diagnosis of chronic condition; exit from study after exposure date. We excluded patients with an AKI diagnosis before index date; or those diagnosed within 6 weeks after exposure, to account for patients with a physiological drop in estimated GFR (eGFR) as a result of the mechanism of RAAS inhibition.

### Variables

#### Exposure

Exposed patients were those with a first ever prescription for RAAS inhibitors or diuretics within the study period (1st January 2008 until 30th September 2015). The date of this first prescription is designated as the index date. Unexposed patients did not have any prescriptions for RAAS inhibitors or diuretics in their medical records; they were matched (1:1) to the exposed patients on age (within 3 years), sex, and time between first chronic condition diagnosis and exposure date (within 6 months). Unexposed patients inherited the index date of the exposed patient they were matched with. Patients had at least 18 months of up-to-standard registration prior to their exposure date, to ensure that exposed patients were new users of the medication and that sufficient baseline covariable information was available. To account for differences in severity of disease between groups, unexposed patients not receiving any medications relevant to their chronic conditions were excluded.

#### Outcomes

Our outcome was AKI, as indicated by relevant medical codes in CPRD GOLD (see code lists) or ICD-10 code N17 in any diagnosis field of a hospital admission in the linked HES data where available.

#### Covariables

Covariables included sex, age at index date, time since diagnosis of first chronic condition, number of types of relevant medications prescribed in the 18 months before index date (e.g. beta-blockers, calcium channel blockers, etc.), number of General Practice (GP) consultations (within 18 months before index date), average systolic blood pressure (within 18 months before index date), smoking status (most recent before index date), kidney function (most recent eGFR before and up to 1 month after index date), and binary flags representing each of the five chronic conditions: CKD, diabetes mellitus, CCF, hypertension, or ischaemic heart disease.

#### Follow-up

Patients were followed up until the earliest of the following: first indication of AKI; death; transfer out from practice; end of practice data collection; or end of the study period (30th September 2015). Date of death was taken from the ONS mortality records where available, and otherwise from CPRD GOLD.

### Statistical analyses

#### Main analyses

To estimate the association between prescription of RAAS inhibitors and/or diuretics and AKI, we conducted multivariable Cox regression, with AKI as our event of interest. Covariables included sex, age, chronic conditions (CKD, diabetes mellitus, CCF, hypertension, ischaemic heart disease), duration of chronic condition, number of medications, number of GP consultations, systolic blood pressure, smoking and GFR. These covariables are conceptualised as confounders due to their association with AKI and potential exposure. As anyone with an AKI within 6 weeks after index date was excluded, we started the time-to-event analysis at 6 weeks after index date. We also investigated possible interactions between exposure and number of medications as such an interaction was found post-hoc in our complete case analyses.

#### Missing covariable data

Some data were missing for smoking status, systolic blood pressure, and renal function (see Table 1). We conducted multiple imputation using chained equations to account for the missing data in these covariables using the ‘ice’ command in Stata. Twenty datasets were imputed, with an imputation model that included the outcome, exposure, and all covariables, as well as any appropriate interaction terms. Additionally, we conducted a complete case analysis to compare our results.

**Table 1:** Covariable information for non-missing data, by exposure (RAAS blockers / diuretics) and outcome (acute kidney injury)

	Exposed (*n* = 70,476)					Unexposed (n = 70,476)				
AKI	Count	%			Missing (%)	Count	%			Missing (%)
AKI	586	0.8			0.0	348	0.5			0.0
No AKI	69,890	99.2			0.0	70,128	99.5			0.0
Gender	Male (%)	Female (%)			Missing (%)	Male (%)	Female (%)			Missing (%)
AKI	358 (61.1)	228 (38.9)			0.0	202 (58.1)	146 (42.0)			0.0
No AKI	38,007 (54.4)	31,883 (45.6)			0.0	38,163 (54.4)	31,965 (45.6)			0.0
Age at Exposure	< 65 (%)	65–74 (%)	> = 75 (%)		Missing (%)	< 65 (%)	65–74 (%)	> = 75 (%)		Missing (%)
AKI	229 (39.1)	179 (30.6)	178 (30.4)		0.0	137 (39.4)	108 (31.0)	103 (29.6)		0.0
No AKI	37,096 (53.1)	19,409 (27.8)	13,385 (19.2)		0.0	36,589 (52.2)	20,331 (29.0)	13,208 (18.8)		0.0
Diagnosis to Exposure	< 30 days (%)	30–179 (%)	180–364 (%)	> = 365 (%)	Missing (%)	< 30 days (%)	30–179 (%)	180–364 (%)	> = 365 (%)	Missing (%)
AKI	191 (32.6)	83 (14.2)	42 (7.2)	270 (46.1)	0.0	39 (11.3)	115 (33.2)	35 (10.1)	157 (45.4)	0.0
No AKI	26,347 (37.7)	8880 (12.7)	4015 (5.7)	30,648 (43.9)	0.0	7203 (10.3)	25,894 (36.9)	5736 (8.2)	31,297 (44.6)	0.0
# Medications	1 (%)	> = 2 (%)			Missing (%)	1 (%)	> = 2 (%)			Missing (%)
AKI	256 (43.7)	330 (56.3)			0.0	297 (85.8)	49 (14.2)			0.0
No AKI	34,803 (49.8)	35,087 (50.2)			0.0	60,292 (86.0)	9838 (14.0)			0.0
# GP Consultation	< 10 (%)	10–19 (%)	20–29 (%)	> = 30 (%)	Missing (%)	< 10 (%)	10–19 (%)	20–29 (%)	> = 30 (%)	Missing (%)
AKI	142 (24.2)	192 (32.8)	126 (21.5)	126 (21.5)	0.0	72 (20.7)	123 (35.3)	74 (21.3)	79 (22.7)	0.0
No AKI	20,781 (29.7)	24,887 (35.6)	12,814 (18.3)	11,408 (16.3)	0.0	18,615 (26.5)	26,116 (37.2)	13,457 (19.2)	11,940 (17.0)	0.0
Systolic Blood Pressure	< 120 (%)	120–139 (%)	140–159 (%)	> = 160 (%)	Missing (%)	< 120 (%)	120–139 (%)	140–159 (%)	> = 160 (%)	Missing (%)
AKI	30 (5.5)	139 (25.4)	226 (41.2)	153 (27.9)	6.5	25 (7.3)	131 (38.3)	150 (43.9)	36 (10.5)	1.7
No AKI	2089 (3.2)	13,458 (20.8)	30,641 (47.3)	18,637 (28.8)	7.2	5202 (7.7)	24,012 (35.6)	28,854 (42.7)	9479 (14.0)	3.7
Smoking	Yes (%)	No (%)	Ex (%)		Missing (%)	Yes (%)	No (%)	Ex (%)		Missing (%)
AKI	115 (19.8)	247 (42.5)	219 (37.7)			71 (20.4)	156 (44.8)	121 (34.8)		0.0
No AKI	12,260 (17.6)	34,823 (50.1)	22,487 (32.3)			12,104 (17.3)	35,521 (50.8)	22,364 (32.0)		0.2
GFR	> = 60 (%)	45–59 (%)	< 45 (%)		Missing (%)	> = 60 (%)	45–59 (%)	< 45 (%)		Missing (%)
AKI	398 (77.7)	81 (15.8)	33 (6.5)			239 (76.1)	55 (17.5)	20 (6.4)		9.8
No AKI	42,103 (85.8)	5883 (12.0)	1070 (2.2)			38,014 (84.9)	6090 (13.6)	678 (1.5)		36.1
# Chronic Conditions	1 (%)	> = 2 (%)			Missing (%)	1 (%)	> = 2 (%)			Missing (%)
AKI	417 (71.2)	169 (28.8)				285 (82.4)	61 (17.6)			0.0
No AKI	56,163 (80.4)	13,727 (19.6)				61,945 (88.3)	8185 (11.7)			0.0

^1^ Percentages exclude missing values, except for the “Missing” column which shows the percentage of patients with missing data

To further address any confounding by indication, we repeated the Cox regression analysis adjusting for continuous propensity scores [17], representing the patients’ propensity for being exposed given the values of other covariables. The propensity score represented the predicted probability of treatment, based on a logistic regression model where exposure status (exposed/unexposed) was regressed against the baseline covariables.

The number needed to treat (NNT) [18, 19] was calculated from the adjusted difference in survival between groups, using the relevant baseline survival probability for the unexposed group (at one, two and 3 years) and appropriate adjusted hazard ratio from the multivariable Cox regression.

#### Sensitivity analyses

To control for potential residual and unmeasured confounding, we carried out a prior event rate ratio analysis (PERR) [20, 21]. For the PERR analysis, two unadjusted Cox regressions were conducted: one to estimate the hazard ratio for AKI in the period before exposure (i.e. differences between the exposed/unexposed groups not due to exposure), and another to estimate the hazard ratio for AKI between the groups after exposure. The PERR result is the ‘after’ hazard ratio divided by the ‘before’ hazard ratio, controlling for any differences between the groups before exposure which might be confounding (whether measured or unmeasured). As such, this analysis required us to construct a slightly different matched dataset, where patients with AKI prior to exposure were not excluded. We required patients to have 3 years of registration prior to exposure, and censored follow-up at 3 years after exposure for the PERR analysis. When checking the assumptions for the PERR analysis, it was evident that the event rate for AKI increased in the 6 months prior to exposure to RAAS inhibitors/ diuretics in patients in the exposed group. A sensitivity analysis was therefore undertaken excluding exposed and un-exposed patients experiencing an AKI episode in that period.

Several additional sensitivity analyses were carried out. These included (1) matching exposed patients to unexposed patients prescribed any other antihypertensive within 6 months of the index date (Additional file 1), (2) excluding patients with strong indications for RAAS inhibition (proteinuric CKD and CCF) (Additional file 2), (3) repeating the analysis in the subset of data linked to HES (which provided information on ethnicity) and quintiles of the Index of Multiple Deprivation (Additional files 3 and 4) comparing the risk of AKI in individuals prescribing RAAS inhibitors or diuretics alone, versus those prescribe both (Additional file 4). All statistical analyses were conducted using Stata 14.

## Results

### Descriptive information

**Fig. 1:**
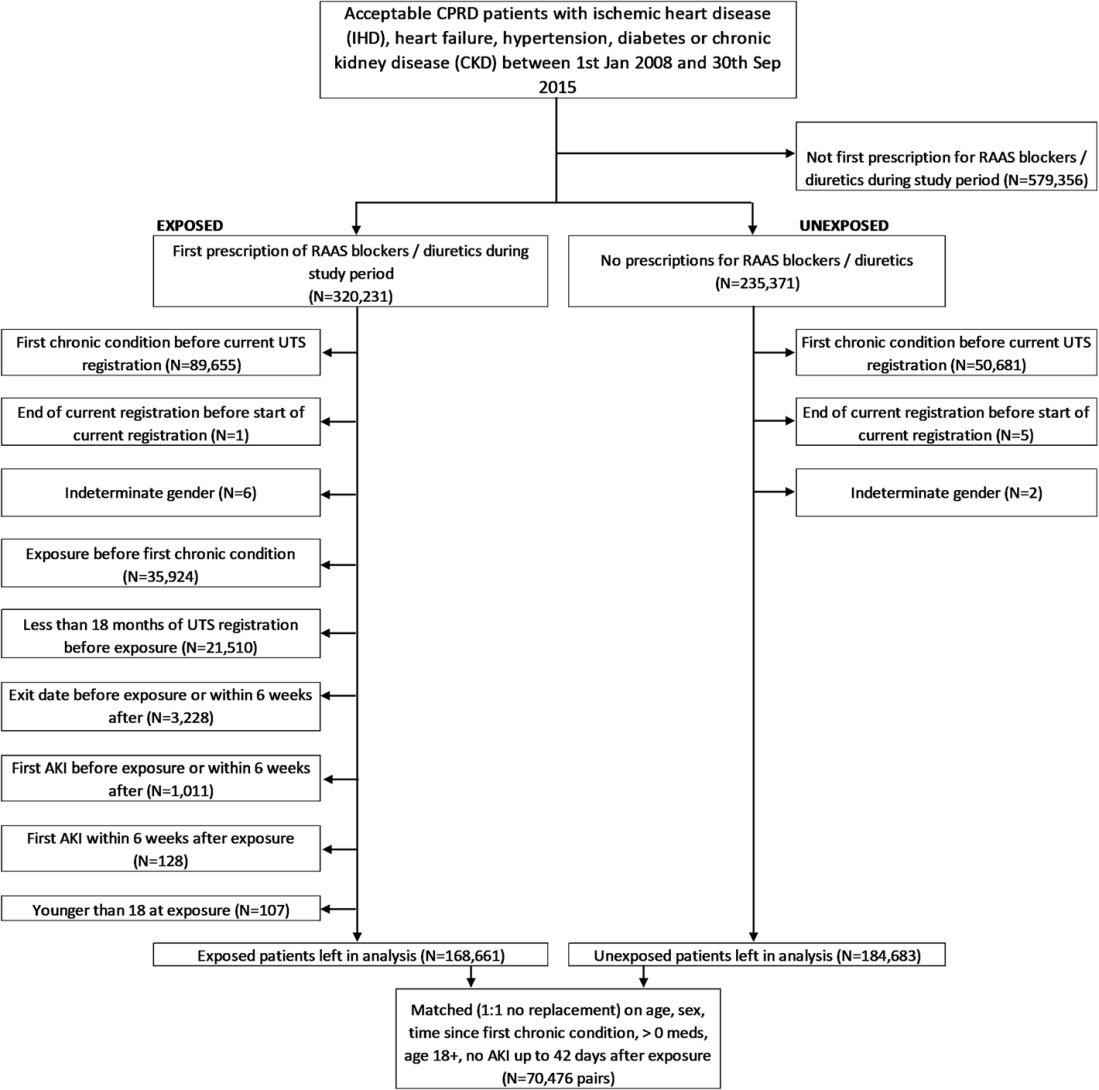
Exclusions for exposed and unexposed cohorts

Between 1st January 2008 and 30th September 2015, 320,231 patients were prescribed their first ever RAAS inhibitor or diuretic. After applying the inclusion/exclusion criteria, 168,661 were available for matching, and 70,476 were matched to unexposed patients (Fig. 1 Exclusions for exposed and unexposed cohorts). Table 1 shows that the exposed and unexposed groups are well matched on age and sex. The exposed group tended to be on more types of medications for their conditions, and were more hypertensive, but were otherwise similar. Most of the missing data was for renal function.

Table 2 shows that rates of AKI (per 1000 person-years) were higher amongst the exposed group than the unexposed group. Rates were generally higher for men, older age groups, people on more types of medication (more notable in the exposed group), people having more GP consultations, those with lower blood pressure (more notable in the exposed group), smokers or ex-smokers, people with worse renal function, and higher for those with a specific chronic condition compared to those without it for all conditions except hypertension.

**Table 2:** Acute kidney injury rates (per 1000 person-years) by covariables (non-missing)

	Exposed (n = 70,476)				Unexposed (n = 70,476)			
Overall	Rate (95% CI)^1^				Rate (95% CI)			
	2.54 (2.34–2.76)				1.7 (1.53–1.89)			
Gender	Male	Female			Male	Female		
	2.84 (2.56–3.15)	2.18 (1.91–2.48)			1.8 (1.57–2.07)	1.57 (1.33–1.84)		
Age at Exposure	< 65	65–74	> = 75		< 65	65–74	> = 75	
	1.82 (1.6–2.08)	2.8 (2.41–3.24)	4.35 (3.75–5.03)		1.22 (1.03–1.45)	1.82 (1.51–2.2)	2.99 (2.46–3.563)	
Diagnosis to Exposure	< 30 days	30–179	180–364	> = 365	< 30 days	30–179	180–364	> = 365
	2.24 (1.95–2.59)	3.04 (2.45–3.77)	3.26 (2.41–4.41)	2.56 (2.28–2.89)	2.21 (1.62–3.03)	1.54 (1.28–1.84)	2.14 (1.54–2.98)	1.65 (1.41–1.93)
# Medications	1	> = 2			1	> = 2		
	2.14 (1.89–2.42)	2.98 (2.67–3.31)			1.71 (1.53–1.92)	1.61 (1.22–2.13)		
# GP Consultation	< 10	10–19	20–29	> = 30	< 10	10–19	20–29	> = 30
	1.9 (1.61–2.24)	2.31 (2–2.66)	3.12 (2.62–3.72)	3.9 (3.27–4.64)	1.18 (0.93–1.49)	1.57 (1.32–1.88)	2.01 (1.6–2.52)	2.71 (2.17–3.37)
Systolic Blood Pressure	< 120	120–139	140–159	> = 160	< 120	120–139	140–159	> = 160
	4.66	3.18	2.19	2.19	1.37	1.77	1.73	1.34
Smoking	Yes	No	Ex		Yes (%)	No (%)	Ex (%)	
	2.63	2.05	3.01		2.09	1.49	1.73	
GFR	> = 60	45–59	< 45		> = 60	45–59	< 45	
	2.3	2.6	7.41		1.54	1.92	5.33	
# Chronic Conditions	1	> = 2			1	> = 2		
	2.27 (2.06–2.50)	3.61 (3.10–4.19)			1.58 (1.40–1.77)	2.64 (2.06–3.40)		

^1^ Numbers in brackets are 95% confidence intervals

### Association between exposure to RAAS inhibitors/ diuretics and acute kidney injury

#### Main analyses

The results for the multivariable adjusted Cox regression (Table 3) and the propensity score adjusted Cox regression (Table 4) showed an increased risk of AKI of 23–24% following exposure to RAAS inhibitors / diuretics: (multivariable cox regression HR = 1.23; 95% CI 1.04–1.45 and propensity score HR = 1.24; 95% CI 1.05–1.47). However, this was qualified by a significant interaction between the exposure and the number of types of medication a patient was prescribed in the 18 months up to exposure (*p*-value for 20 imputed datasets ranged between 0.0186 and 0.023. It appears that any additional risk of AKI following RAAS inhibitors / diuretics occurs in patients already taking other types of medications. The numbers needed to treat with RAAS inhibitors/diuretics to observe one additional AKI event are presented in Appendix 1.

**Table 3:** Cox Regression Models (*n* = 140,952)

Model	Covariables	HR (AKI)	95% LCI	95% UCI
Baseline^1^	*Unexposed*	1		
	*Exposed*	1.48	1.3	1.7
Baseline + Sex	*Unexposed*	1		
	*Exposed*	1.48	1.3	1.7
	*Male*	1		
	*Female*	0.81	0.71	0.92
Baseline + Age	*Unexposed*	1		
	*Exposed*	1.48	1.29	1.69
	*< 65 years*	1		
	*65–74*	1.53	1.31	1.79
	*> = 75*	2.46	2.11	2.88
Baseline + Chronic Time	*Unexposed*	1		
	*Exposed*	1.53	1.33	1.77
	*< 30 days*	1		
	*30–179 days*	1.1	0.89	1.35
	*180–364 days*	1.37	1.05	1.78
	*> = 365 days*	1.05	0.89	1.24
Baseline + CKD	*Unexposed*	1		
	*Exposed*	1.5	1.32	1.72
	*No CKD*	1		
	*CKD*	1.93	1.63	2.27
Baseline + DM	*Unexposed*	1		
	*Exposed*	1.41	1.24	1.61
	*No DM*	1		
	*DM*	1.89	1.61	2.21
Baseline + HF	*Unexposed*	1		
	*Exposed*	1.41	1.23	1.61
	*No HF*	1		
	*HF*	3.65	2.74	4.86
Baseline + HT	*Unexposed*	1		
	*Exposed*	1.59	1.39	1.82
	*No HT*	1		
	*HT*	0.53	0.47	0.61
Baseline + IHD	*Unexposed*	1		
	*Exposed*	1.5	1.31	1.71
	*No IHD*	1		
	*IHD*	1.17	1	1.37
Baseline + Medications	*Unexposed*	1		
	*Exposed*	1.37	1.19	1.58
	*1*	1		
	*> = 2*	1.27	1.11	1.46
Baseline + GP Consultations	*Unexposed*	1		
	*Exposed*	1.5	1.31	1.71
	*< 10*	1		
	*10–19*	1.27	1.07	1.51
	*20–29*	1.71	1.41	2.07
	*> = 30*	2.23	1.84	2.71
Baseline + SBP	*Unexposed*	1		
	*Exposed*	1.59	1.38	1.83
	*< 120*	1		
	*120–139*	0.88	0.65	1.17
	*140–159*	0.71	0.53	0.94
	*> = 160*	0.68	0.5	0.92
Baseline + Smoking	*Unexposed*	1		
	*Exposed*	1.48	1.3	1.69
	*No*	1		
	*Yes*	1.35	1.13	1.6
	*Ex*	1.34	1.16	1.54
Baseline + GFR	*Unexposed*	1		
	*Exposed*	1.47	1.29	1.68
	*> = 60*	1		
	*45–59*	1.29	1.07	1.55
	*< 45*	2.92	2.2	3.88
Full Model	*Unexposed*	1		
	*Exposed*	1.23	1.04	1.45
Full Model (inc meds*exposure)	*1 (exposed)*	1.1	0.91	1.33
	*> = 2 (exposed)*	1.49	1.06	2.11

^1^ “Baseline” – hazard ratio prior to adjustment for covariable(s)

**Table 4:** Cox Regression Models adjusted by Propensity Scores for Disease Severity^1^ (*n* = 140,952)

Model	Covariates	HR (AKI)	95% LCI	95% UCI
Baseline	*Unexposed*	1		
	*Exposed*	1.48	1.3	1.7
Baseline + P-Score (Full Model)	*Unexposed*	1		
	*Exposed*	1.24	1.05	1.47
Full Model (inc meds*exposure)	*1 (exposed)*	1.09	0.89	1.33
	*> = 2 (exposed)*	1.61	1.14	2.28

^1^ Variables in the propensity score model were: gender, age, time since first chronic condition, number of medications, number of GP consultations, chronic condition flags, systolic blood pressure, kidney function (GFR), and smoking status

#### Sensitivity analyses

**Fig. 2:**
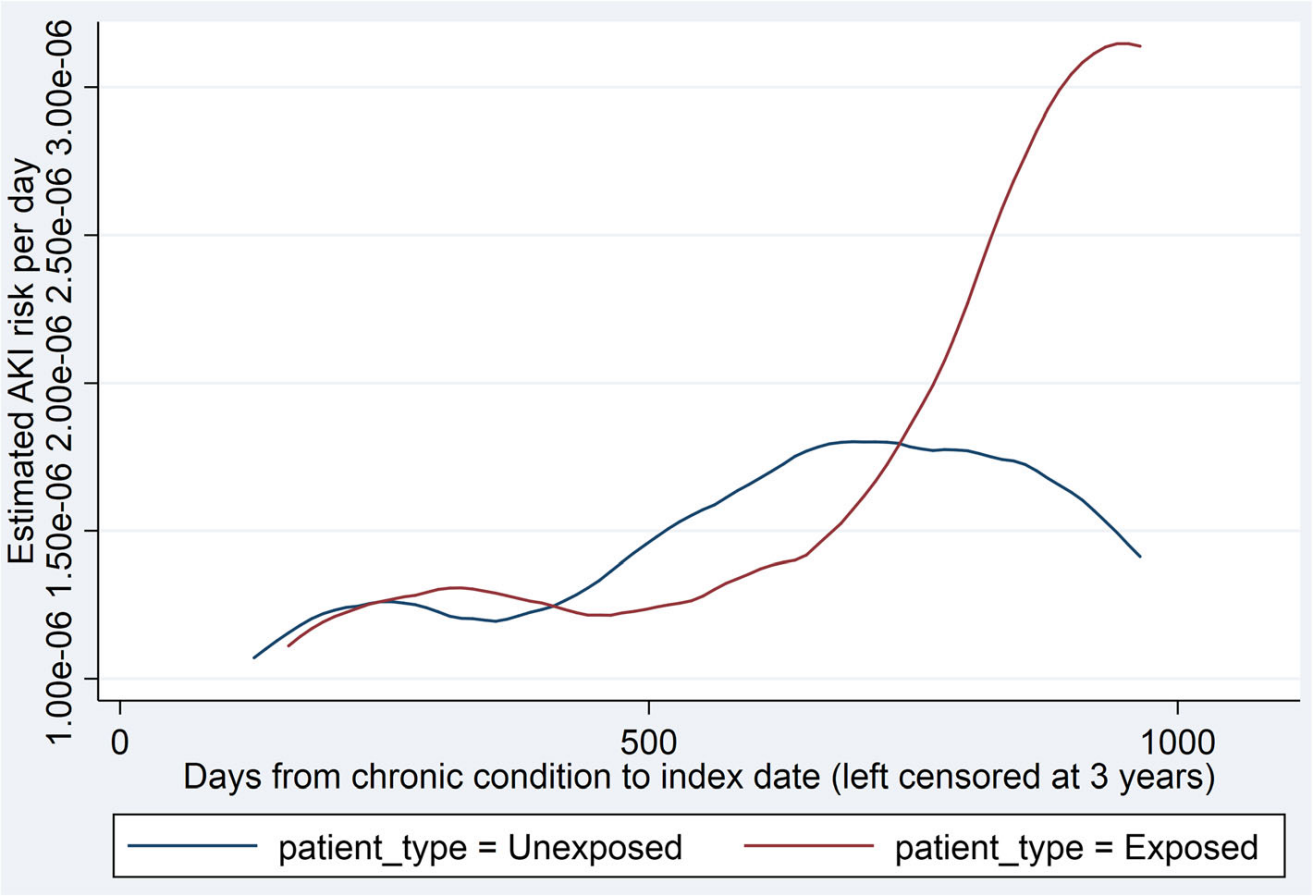
Acute kidney injury hazards in time before exposure

The PERR analysis, which additionally adjusts for unmeasured confounding, provided a similar overall hazard ratio to the main analysis with a wider confidence interval (HR = 1.29; 95% CI 0.94–1.63). AKI rates in the two groups were not proportional in the period leading up to exposure, with a notable increase in the AKI rate in the exposed group in the 6 months prior to exposure (Fig. 2 Acute kidney injury hazards in time before exposure.). Non-proportionality was greatest in those with the longest duration of diagnosis of chronic condition. A sensitivity analysis was conducted excluding patients with AKI in the 6 months prior to exposure. This resulted in a higher PERR estimate (HR = 1.85; 95% CI 0.91–2.79), however confidence intervals were wide.

Results for the complete case analyses were similar to those using imputed data (data not shown). Analyses restricted to the subset of data linked to HES which provided information on ethnicity and quintiles of the Index of Multiple Deprivation gave similar results to the main analyses (Additional file 3).

## Discussion

This study demonstrated a significantly increased risk of AKI in individuals taking RAAS inhibitors or diuretics in the presence of any additional antihypertensive medication. There was, however, no association between AKI risk and the number of antihypertensive medications in the unexposed group. Despite a significantly increased relative risk of AKI attributable to RAAS inhibition/diuretic use in the exposed group, the absolute risk of AKI in these individuals was small. Further, as demonstrated in the PERR analysis, we observed an increasing risk of AKI in the months preceding initiation of RAAS inhibition which has not previously been reported: Increased risk in the exposed group was seen in those with the longest duration of diagnosis of chronic condition. This could be explained by a worsening of clinical condition warranting initiation of further medication. Alternatively, it could imply reverse causation whereby having an AKI episode results in an increased likelihood of exposure to RAAS inhibitors or diuretics, which needs consideration in future observational studies of AKI risk.

The association between RAAS inhibitor/diuretic use and AKI risk demonstrated in this study is consistent with previous literature [10, 11, 22] and can be explained biologically. RAAS inhibitors reduce tone in the efferent arteriole of the glomerulus and thus impair renal autoregulation, maintenance of intraglomerular pressure and glomerular filtration. Without directly targeting the RAAS system, medication that lowers pre-glomerular pressure by reducing the effective circulating volume either by volume contraction (i.e. diuretics) is also likely to increase the risk of AKI [23]. Glomerular filtration pressure can also be very dependent on RAAS-mediated chronic tone in the efferent arteriole in people with CCF or renal artery stenosis, and those with disruptions to the renal microcirculation, [24] making them further sensitive to such effects.

Although this study demonstrated an increased risk of AKI with RAAS inhibition/ diuretic use, the absolute risk of AKI in the exposed group was low (2.5 and 1.7 events per 1000 person years in people taking RAAS inhibitors/ diuretics and those not, respectively). With a small or even moderate effect of RAAS inhibition/diuretic on the risk of AKI the NNT will therefore be high. In the subset of individuals with a creatinine >124umol/L included in the HOPE randomised trial [25], the NNT with Ramipril to prevent one myocardial infarction or one stroke over 4.5 years was 20.4 and 16.1 respectively. In the Acute Infarction Ramipril Efficacy study, a NNT of 17.5 over 6–15 months was found to prevent all-cause mortality in patients with heart failure following myocardial infarction [26]. There is therefore substantial evidence to support a beneficial impact of RAAS inhibition in the prevention of ischaemic heart disease, CCF, cerebrovascular disease, proteinuric CKD, ESKD, all-cause and cardiovascular mortality [25–28]. As a result, RAAS inhibitors have become the second most commonly prescribed medication in General Practice (GP) in the UK, accounting for 6% of all prescriptions [29].

Given this, any public health or individual patient level recommendations about the initiation or temporary/ permanent discontinuation of RAAS inhibitors/ diuretics must consider both the potential risk of harm as well as benefit. The risk of AKI associated with RAAS inhibition must be weighed against the potential benefits for each individual, including delayed progression of CKD, [30] reduction in cardiovascular events (myocardial infarction, cerebrovascular events, resistant heart failure following myocardial infarction) and mortality (all-cause and cardiovascular-specific) [25, 26, 31]. The risk benefit ratio is likely to be influenced by level of comorbidity as well as concurrent medications [10]. In the general population of the UK, the benefits of RAAS inhibition are likely to outweigh the disadvantages in terms of AKI. However, when prescribing RAAS inhibitors and diuretics, physicians should be aware that the risk of AKI increases with number of additional hypertensive medications and diuretic use. The mechanism of the increased risk is likely to relate to actual or relative reduction in effective circulating volume; volume contraction and hypotension should therefore be avoided.

This study has several strengths and limitations. The size of the cohort and geographical spread of recruitment should enable the results to be generalisable to all primary care populations in England. The exposed and unexposed groups were well-matched, reducing bias, and the use of multiple statistical models with similar results increase confidence in the effect estimate. In addition, the results of this study support the majority of existing literature in this area. However, the diagnosis of AKI was based upon HES data/READ codes rather than serum biochemistry, and AKI rates will therefore be underestimated. The exclusion of individuals with an AKI event prior to the index date or within 6 weeks of initiation of RAAS inhibitors or diuretics was designed to minimise baseline differences in AKI risk between groups. However by doing so we may have inadvertently excluded those with a higher risk of AKI. Medication dose and dose changes were not explored in this analysis. This may be of particular relevance to RAAS inhibitors and diuretics where a dose-dependent impact on renal function may be seen, and physiological changes in measured function may therefore be interpreted as AKI events. Further alternative explanations for our findings include the possibility of increased testing of serum biochemistry in the exposed group resulting in higher detection of AKI. As with most observational studies there is the possibility of residual confounding in the Cox and propensity-matched analyses though this should have been less of an issue in the PERR analysis.

## Conclusions

This study supports previous literature regarding an increased risk of AKI in individuals prescribed RAAS inhibitors and diuretics [10, 11, 22]. However, the results suggest the absolute increased risk in AKI is low and limited to patients prescribed concurrent antihypertensive medications. Further, the absolute risk of AKI associated with RAAS inhibitors and diuretics is low and likely to be significantly outweighed by the multitude of beneficial effects demonstrated from RAAS inhibition in the literature, [25, 30, 31] . Given that the impact of sick day rules on patients’ adherence to RAAS inhibitor/ diuretic treatment is unknown, interventional studies are required to inform practice that will optimise all patient outcomes, not just AKI-related ones.

### Supplementary information

**Additional file 1.** Unexposed patients matched (1:1) to exposed patients on age (within 3 years), sex and time between prescription of any other antihypertensive medication and exposure date (within 6 months). A sensitivity analysis.

**Additional file 2.** Excluding patients with strong indication for renin-angiotensin-aldosterone blockade (proteinuric chronic kidney disease and congestive cardiac failure). A sensitivity analysis.

**Additional file 3.** Analyses limited to those patients eligible for linkage to Hospital Episode Statistics and quintiles of the Index of Multiple Deprivation. A sensitivity analysis.

**Additional file 4. **Sensitivity analysis comparing individuals prescribed either RAAS inhibitors or diuretics alone, with those receiving both classes of medication**.**

## Data Availability

The data that support the findings of this study are available from CPRD (www.CPRD.com) but restrictions apply to the availability of these data, which were used under license for the current study, and so are not publicly available. Data are however available from the authors upon reasonable request and with permission of CPRD. Code lists are available from [https://github.com/jonestim2002/aki_raas_diuretics].

## Appendix

**Table 5:** Numbers needed to treat with renin angiotensin aldosterone inhibitors or diuretics to observe one additional acute kidney injury event

		NNT
1 medication	One year	6951
	Three years	2370
2+ medications	One year	966
	Three years	336

## Abbreviations

(e)GFR: (estimated) Glomerular Filtration Rate
AKI: Acute kidney injury
CCF: Congestive cardiac failure
CI: Confidence interval
CKD: Chronic kidney disease
CPRD: Clinical Practice Research Datalink
ESKD: End-stage kidney disease
GP: General Practice
HES-APC: Hospital Episode Statistics – Admitted Patient Records
HR: Hazard ratio
ICD-10: International Classification of Diseases (10)
NNT: Number needed to treat
NSAID: Non-steroidal anti-inflammatory drug
ONS: Office of National Statistics
PERR: Prior event rate ratio
RAAS: Renin angiotensin aldosterone system
UK: United Kingdom
