# Supplementary material for: Estimating the risk of acute kidney injury associated with use of diuretics and renin angiotensin aldosterone system inhibitors: A population based cohort study using the clinical practice research datalink
Source: BMC Nephrol. 2019 Dec 30;20:481. doi: 10.1186/s12882-019-1633-2 (PMC6937998; doi:10.1186/s12882-019-1633-2)
Supplement: Supplementary file 1 — Additional file 1. Unexposed patients matched (1:1) to exposed patients on age (within 3 years), sex and time between prescription of any other antihypertensive medication and exposure date (within 6 months). A sensitivity analysis. [file 12882_2019_1633_MOESM1_ESM.docx]

| **Table 1. Covariable information for non-missing data, by exposure (RAAS blockers / diuretics) and outcome (acute kidney injury)**  **Additional File 1.**  **Sensitivity analysis: Unexposed patients matched (1:1) to exposed patients on age (within 3 years), sex and time between prescription of any other antihypertensive medication and exposure date (within 6 months).** | | | | | | | | | | | | |
| --- | --- | --- | --- | --- | --- | --- | --- | --- | --- | --- | --- | --- |
|  |  |  |  |  |  |  |  |  |  |  |  |  |
|  |  | **Exposed (n=39,586)** | | | | |  | **Unexposed (n=39,586)** | | | | |
|  |  |  |  |  |  |  |  |  |  |  |  |  |
| **AKI** |  | *Count* | *%* |  |  | *Missing (%)* |  | *Count* | *%* |  |  | *Missing (%)* |
| AKI |  | 297 | 0.8 |  |  | 0.0 |  | 170 | 0.4 |  |  | 0.0 |
| No AKI |  | 39,289 | 99.2 |  |  | 0.0 |  | 39,416 | 99.6 |  |  | 0.0 |
| **Gender** |  | *Male* | *Female* |  |  | *Missing (%)* |  | *Male* | *Female* |  |  | *Missing (%)* |
| AKI |  | 183 (61.6) | 114 (38.4) |  |  | 0.0 |  | 108 (63.5) | 62 (36.5) |  |  | 0.0 |
| No AKI |  | 21,937 (55.8) | 17,352 (44.2) |  |  | 0.0 |  | 22,012 (55.8) | 17,404 (44.2) |  |  | 0.0 |
| **Age at Exposure** |  | *<65* | *65-74* | *>=75* |  | *Missing (%)* |  | *<65* | *65-74* | *>=75* |  | *Missing (%)* |
| AKI |  | 115 (38.7) | 102 (34.3) | 80 (26.9) |  | 0.0 |  | 73 (42.9) | 57 (33.5) | 40 (23.5) |  | 0.0 |
| No AKI |  | 21,236 (54.1) | 11,437 (29.1) | 6,616 (16.8) |  | 0.0 |  | 21,073 (53.5) | 11,812 (30) | 6,531 (16.6) |  | 0.0 |
| **Diagnosis to Exposure** |  | *< 30 days* | *30-179* | *180-364* | *>= 365* | *Missing (%)* |  | *< 30 days* | *30-179* | *180-364* | *>= 365* | *Missing (%)* |
| AKI |  | 126 (42.4) | 57 (19.2) | 13 (4.4) | 101 (34) | 0.0 |  | 40 (23.5) | 23 (13.5) | 11 (6.5) | 53 (31.2) | 0.0 |
| No AKI |  | 18,771 (47.8) | 6,166 (15.7) | 2,092 (5.3) | 12,260 (31.2) | 0.0 |  | 11,138 (28.3) | 4,433 (11.2) | 1,617 (4.1) | 12,318 (31.3) | 0.0 |
| **# Medications** |  | *1 (%)* | *>= 2 (%)* |  |  | *Missing (%)* |  | *1 (%)* | *>= 2 (%)* |  |  | *Missing (%)* |
| AKI |  | 128 (43.1) | 169 (56.9) |  |  | 0.0 |  | 132 (77.6) | 38 (22.4) |  |  | 0.0 |
| No AKI |  | 20,280 (51.6) | 19,009 (48.4) |  |  | 0.0 |  | 31,800 (80.7) | 7,616 (19.3) |  |  | 0.0 |
| **# GP Consultations** |  | *<10* | *10-19* | *20-29* | *>=30* | *Missing (%)* |  | *<10* | *10-19* | *20-29* | *>=30* | *Missing (%)* |
| AKI |  | 79 (26.6) | 86 (29) | 71 (23.9) | 61 (20.5) | 0.0 |  | 45 (26.5) | 70 (41.2) | 25 (14.7) | 30 (17.6) | 0.0 |
| No AKI |  | 12,355 (31.4) | 14,056 (35.8) | 6,973 (17.7) | 5,905 (15) | 0.0 |  | 14,080 (35.7) | 13,418 (34) | 6,468 (16.4) | 5,450 (13.8) | 0.0 |
| **Systolic Blood Pressure** |  | < 120 | 120-139 | 140-159 | >=160 | *Missing (%)* |  | < 120 | 120-139 | 140-159 | >=160 | *Missing (%)* |
| AKI |  | 15 (5.5) | 56 (20.5) | 127 (46.5) | 75 (27.5) | 8.1 |  | 9 (5.5) | 48 (29.4) | 70 (42.9) | 36 (22.1) | 4.1 |
| No AKI |  | 1,172 (3.2) | 6,818 (18.8) | 16,703 (46.1) | 11,539 (31.8) | 7.8 |  | 1,752 (4.9) | 8,160 (22.9) | 15,848 (44.6) | 9,804 (27.6) | 9.8 |
| **Smoking** |  | *Yes (%)* | *No (%)* | *Ex (%)* |  | *Missing (%)* |  | *Yes (%)* | *No (%)* | *Ex (%)* |  | *Missing (%)* |
| AKI |  | 60 (20.3) | 111 (37.6) | 124 (42) |  | 0.7 |  | 38 (22.6) | 75 (44.6) | 55 (32.7) |  | 1.2 |
| No AKI |  | 7,066 (18.1) | 19,444 (49.8) | 12,564 (32.2) |  | 0.5 |  | 7,055 (18) | 19,974 (50.9) | 12,201 (31.1) |  | 0.5 |
| **GFR** |  | *>= 60* | *45-59* | *< 45* |  | *Missing (%)* |  | *>= 60* | *45-59* | *< 45* |  | *Missing (%)* |
| AKI |  | 200 (78.1) | 39 (15.2) | 17 (6.6) |  | 13.8 |  | 121 (81.8) | 19 (12.8) | 8 (5.4) |  | 12.9 |
| No AKI |  | 23,861 (87) | 3,077 (11.2) | 493 (1.8) |  | 30.2 |  | 22,471 (86.6) | 3,143 (12.1) | 337 (1.3) |  | 34.2 |
| **# Chronic Conditions** |  | *1 (%)* | *>= 2 (%)* |  |  | *Missing (%)* |  | *1 (%)* | *>= 2 (%)* |  |  | *Missing (%)* |
| AKI |  | 231 (77.8) | 66 (22.2) |  |  | 0.0 |  | 150 (88.2) | 20 (11.8) |  |  | 0.0 |
| No AKI |  | 33,245 (84.6) | 6,044 (15.4) |  |  | 0.0 |  | 35,837 (90.9) | 3,579 (9.1) |  |  | 0.0 |
| **Chronic Conditions** |  | *Count* | *%* |  |  |  |  | *Count* | *%* |  |  |  |
| Chronic Kidney Disease |  | 3,346 | 8.5 |  |  |  |  | 3,855 | 9.7 |  |  |  |
| Diabetes |  | 5,023 | 12.7 |  |  |  |  | 3,387 | 8.6 |  |  |  |
| Heart Failure |  | 1,393 | 3.5 |  |  |  |  | 128 | 0.3 |  |  |  |
| Hypertension |  | 30,404 | 76.8 |  |  |  |  | 29,844 | 75.4 |  |  |  |
| Ischaemic Heart Disease |  | 6,105 | 15.4 |  |  |  |  | 6,188 | 15.6 |  |  |  |

*Percentages exclude missing values, except for the “Missing” column which shows the percentage of patients with missing data.*

| **Table 2. Acute kidney injury rates (per 1,000 person-years) by covariables (non-missing)** | | | | | | | | | | | | | | | | |
| --- | --- | --- | --- | --- | --- | --- | --- | --- | --- | --- | --- | --- | --- | --- | --- | --- |
|  |  |  | |  | |  | |  |  |  | |  | |  | |  |
|  |  | **Exposed (n=39,586)** | | | | | | |  | **Unexposed (n=39,586)** | | | | | | |
|  |  |  |  | |  | |  | |  |  |  | |  | |  | |
| **Overall** |  | *Rate (95% CI)* |  | |  | |  | |  | *Rate (95% CI)* |  | |  | |  | |
|  |  | 2.35 (2.1-2.63) |  | |  | |  | |  | 1.41 (1.21-1.64) |  | |  | |  | |
| **Gender** |  | *Male* | *Female* | |  | |  | |  | *Male* | *Female* | |  | |  | |
|  |  | 2.57 (2.23-2.98) | 2.06 (1.71-2.47) | |  | |  | |  | 1.6 (1.32-1.93) | 1.18 (0.92-1.51) | |  | |  | |
| **Age at Exposure** |  | *<65* | *65-74* | | *>=75* | |  | |  | *<65* | *65-74* | | *>=75* | |  | |
|  |  | 1.63 (1.36-1.96) | 2.8 (2.3-3.4) | | 4.09 (3.28-5.09) | |  | |  | 1.09 (0.87-1.38) | 1.6 (1.23-2.07) | | 2.21 (1.62-3.01) | |  | |
| **Diagnosis to Exposure** |  | *< 30 days* | *30-179* | | *180-364* | | *>= 365* | |  | *< 30 days* | *30-179* | | *180-364* | | *>= 365* | |
|  |  | 2.02 (1.7-2.41) | 2.98 (2.29-3.86) | | 1.98 (1.15-3.4) | | 2.63 (2.16-3.19) | |  | 1.17 (0.86-1.6) | 1.58 (1.04-2.4) | | 2.23 (1.23-4.02) | | 1.43 (1.09-1.88) | |
| **# Medications** |  | *1* | *>= 2* | |  | |  | |  | *1* | *>= 2* | |  | |  | |
|  |  | 1.86 (1.57-2.22) | 2.92 (2.51-3.4) | |  | |  | |  | 1.37 (1.16-1.63) | 1.57 (1.14-2.16) | |  | |  | |
| **# GP Consultations** |  | *<10* | *10-19* | | *20-29* | | *>=30* | |  | *<10* | *10-19* | | *20-29* | | *>=30* | |
|  |  | 1.82 (1.46-2.27) | 1.89 (1.53-2.33) | | 3.35 (2.66-4.23) | | 3.75 (2.92-4.82) | |  | 0.96 (0.72-1.29) | 1.71 (1.35-2.16) | | 1.34 (0.91-1.99) | | 2.1 (1.47-3.01) | |
| **Systolic Blood Pressure** |  | < 120 | 120-139 | | 140-159 | | >=160 | |  | < 120 | 120-139 | | 140-159 | | >=160 | |
|  |  | 4.65 (2.8-7.72) | 2.74 (2.11-3.57) | | 2.34 (1.97-2.78) | | 1.92 (1.53-2.4) | |  | 1.67 (0.87-3.21) | 1.93 (1.45-2.56) | | 1.45 (1.15-1.84) | | 1.19 (0.86-1.65) | |
| **Smoking** |  | *Yes (%)* | *No (%)* | | *Ex (%)* | |  | |  | *Yes (%)* | *No (%)* | | *Ex (%)* | |  | |
|  |  | 2.62 (2.03-3.37) | 1.77 (1.47-2.13) | | 3.11 (2.6-3.7) | |  | |  | 1.77 (1.29-2.43) | 1.22 (0.97-1.53) | | 1.49 (1.14-1.94) | |  | |
| **GFR** |  | *>= 60* | *45-59* | | *< 45* | |  | |  | *>= 60* | *45-59* | | *< 45* | |  | |
|  |  | 2.52 (2.19-2.89) | 3.54 (2.59-4.85) | | 10.71 (6.66-17.23) | |  | |  | 1.67 (1.4-2) | 1.87 (1.19-2.93) | | 8.46 (4.23-16.92) | |  | |
| **# Chronic Conditions** |  | *1* | *>= 2* | |  | |  | |  | *1* | *>= 2* | |  | |  | |
|  |  | 2.16 (1.9-2.45) | 3.4 (2.67-4.33) | |  | |  | |  | 1.37 (1.17-1.61) | 1.83 (1.17-2.87) | |  | |  | |
| **Chronic Conditions** |  | *No* | *Yes* | |  | |  | |  | *No* | *Yes* | |  | |  | |
| Chronic Kidney Disease |  | 2.17 (1.92-2.46) | 4.33 (3.23-5.79) | |  | |  | |  | 1.34 (1.13-1.57) | 2.16 (1.45-3.22) | |  | |  | |
| Diabetes |  | 2.2 (1.95-2.5) | 3.39 (2.58-4.45) | |  | |  | |  | 1.3 (1.11-1.53) | 2.74 (1.85-4.06) | |  | |  | |
| Heart Failure |  | 2.22 (1.97-2.5) | 6.65 (4.46-9.93) | |  | |  | |  | 1.42 (1.22-1.65) | no data | |  | |  | |
| Hypertension |  | 3.81 (3.14-4.63) | 1.96 (1.7-2.25) | |  | |  | |  | 2.14 (1.67-2.73) | 1.17 (0.97-1.42) | |  | |  | |
| Ischaemic Heart Disease |  | 2.23 (1.96-2.53) | 3.05 (2.35-3.96) | |  | |  | |  | 1.36 (1.15-1.61) | 1.67 (1.19-2.34) | |  | |  | |

*Numbers in brackets are 95% confidence intervals*

| **Table 3. Cox Regression Models (n= 79,172)** | | |  |  |
| --- | --- | --- | --- | --- |
|  |  |  |  |  |
| **Model** | **Covariates** | **HR (AKI)** | **95% LCI** | **95% UCI** |
| Baseline^1^ | *Unexposed* | 1 |  |  |
|  | *Exposed* | 1.65 | 1.37 | 2 |
| Baseline + Sex | *Unexposed* | 1 |  |  |
|  | *Exposed* | 1.65 | 1.37 | 2 |
|  | *Male* | 1 |  |  |
|  | *Female* | 0.78 | 0.64 | 0.94 |
| Baseline + Age | *Unexposed* | 1 |  |  |
|  | *Exposed* | 1.65 | 1.37 | 2 |
|  | *< 65 years* | 1 |  |  |
|  | *65-74* | 1.63 | 1.32 | 2.02 |
|  | *>=75* | 2.38 | 1.89 | 2.99 |
| Baseline + Chronic_Time | *Unexposed* | 1 |  |  |
|  | *Exposed* | 1.63 | 1.34 | 1.97 |
|  | *< 30 days* | 1 |  |  |
|  | *30 - 179 days* | 1.28 | 0.98 | 1.69 |
|  | *180 - 364 days* | 1.22 | 0.81 | 1.85 |
|  | *>= 365 days* | 1.2 | 0.98 | 1.48 |
| Baseline + CKD | *Unexposed* | 1 |  |  |
|  | *Exposed* | 1.67 | 1.38 | 2.01 |
|  | *No CKD* | 1 |  |  |
|  | *CKD* | 1.86 | 1.44 | 2.4 |
| Baseline + DM | *Unexposed* | 1 |  |  |
|  | *Exposed* | 1.6 | 1.33 | 1.94 |
|  | *No DM* | 1 |  |  |
|  | *DM* | 1.72 | 1.35 | 2.2 |
| Baseline + HF | *Unexposed* | 1 |  |  |
|  | *Exposed* | 1.58 | 1.3 | 1.92 |
|  | *No HF* | 1 |  |  |
|  | *HF* | 2.95 | 1.95 | 4.47 |
| Baseline + HT | *Unexposed* | 1 |  |  |
|  | *Exposed* | 1.71 | 1.41 | 2.06 |
|  | *No HT* | 1 |  |  |
|  | *HT* | 0.52 | 0.43 | 0.63 |
| Baseline + IHD | *Unexposed* | 1 |  |  |
|  | *Exposed* | 1.67 | 1.38 | 2.01 |
|  | *No IHD* | 1 |  |  |
|  | *IHD* | 1.31 | 1.04 | 1.65 |
| Baseline + Medications | *Unexposed* | 1 |  |  |
|  | *Exposed* | 1.5 | 1.23 | 1.83 |
|  | *1* | 1 |  |  |
|  | *>= 2* | 1.44 | 1.2 | 1.75 |
| Baseline + GP Consultations | *Unexposed* | 1 |  |  |
|  | *Exposed* | 1.62 | 1.34 | 1.96 |
|  | *<10* | 1 |  |  |
|  | *10-19* | 1.3 | 1.03 | 1.65 |
|  | *20-29* | 1.76 | 1.35 | 2.3 |
|  | *>=30* | 2.22 | 1.69 | 2.92 |
| Baseline + SBP | *Unexposed* | 1 |  |  |
|  | *Exposed* | 1.72 | 1.42 | 2.07 |
|  | *<120* | 1 |  |  |
|  | *120-139* | 0.8 | 0.52 | 1.25 |
|  | *140-159* | 0.63 | 0.42 | 0.96 |
|  | *>=160* | 0.51 | 0.33 | 0.79 |
| Baseline + Smoking | *Unexposed* | 1 |  |  |
|  | *Exposed* | 1.65 | 1.35 | 2.08 |
|  | *No* | 1 |  |  |
|  | *Yes* | 1.47 | 1.15 | 1.88 |
|  | *Ex* | 1.54 | 1.25 | 1.89 |
| Basline + GFR | *Unexposed* | 1 |  |  |
|  | *Exposed* | 1.63 | 1.35 | 1.97 |
|  | *>=60* | 1 |  |  |
|  | *45-59* | 1.22 | 0.94 | 1.59 |
|  | *< 45* | 3.34 | 2.23 | 5 |
| Full Model | *Unexposed* | 1 |  |  |
|  | *Exposed* | 1.44 | 1.18 | 1.76 |
| Full Model (inc meds*exposure) | *1 (exposed)* | 1.29 | 1 | 1.65 |
|  | *>= 2 (exposed)* | 1.4 | 0.91 | 2.16 |

*^1^“Baseline” – hazard ratio prior to adjustment for covariable(s).*

| **Table 4. Cox Regression Models adjusted by Propensity Scores for Disease Severity (n = 79,172)** | | | | | |
| --- | --- | --- | --- | --- | --- |
|  |  |  |  |  |  |
|  |  |  |  |  |  |
| **Model** | **Covariates** | **HR (AKI)** | **95% LCI** | **95% UCI** |  |
| Baseline | *Unexposed* | 1 |  |  |  |
|  | *Exposed* | 1.65 | 1.37 | 2 |  |
| Baseline + P-Score (Full Model) | *Unexposed* | 1 |  |  |  |
|  | *Exposed* | 1.45 | 1.18 | 1.77 |  |
| Full Model (inc meds*exposure) | *1 (exposed)* | 1.3 | 1.01 | 1.66 |  |
|  | *>= 2 (exposed)* | 1.41 | 0.92 | 2.16 |  |
| ***Notes*** |  |  |  |  |  |
| *Variables in the propensity score model were: gender, age, time since first chronic condition, number of medications, number of GP consultations, chronic condition flags, systolic blood pressure, kidney function (GFR), and smoking status* | | | | |  |

| **Table 5. Prior-Event-Rate-Ratio (PERR) Analysis.** | | |  |
| --- | --- | --- | --- |
|  |  |  |  |
| *With left truncation at 3 years and excluding those with no days before exposure or AKI within 42 days; right truncation at 3 years (n=43,430)* | | | |
| **HR (prior)** | **HR (post)** | **PERR** |  |
| 0.98 (0.57-1.71) | 2.57 (1.76-3.77) | 2.62 (0.93-4.30)* |  |
|  |  |  |  |
| ** Bootstrapped confidence interval; reps=100* | | |  |
