# Supplementary material for: Estimating the risk of acute kidney injury associated with use of diuretics and renin angiotensin aldosterone system inhibitors: A population based cohort study using the clinical practice research datalink
Source: BMC Nephrol. 2019 Dec 30;20:481. doi: 10.1186/s12882-019-1633-2 (PMC6937998; doi:10.1186/s12882-019-1633-2)
Supplement: Supplementary file 2 — Additional file 2. Excluding patients with strong indication for renin-angiotensin-aldosterone blockade (proteinuric chronic kidney disease and congestive cardiac failure). A sensitivity analysis. [file 12882_2019_1633_MOESM2_ESM.docx]

| **Table 1. Covariable information for non-missing data, by exposure (RAAS blockers / diuretics) and outcome (acute kidney injury)**  **Additional File 2.**  **Sensitivity analysis: Excluding patients with strong indication for renin-angiotensin-aldosterone blockade (proteinuric chronic kidney disease and congestive cardiac failure)** | | | | | | | | | | | |  |
| --- | --- | --- | --- | --- | --- | --- | --- | --- | --- | --- | --- | --- |
|  |  |  |  |  |  |  |  |  |  |  |  |  |
|  |  | **Exposed (n=69,610)** | | | | |  | **Unexposed (n=69,610)** | | | | |
|  |  |  |  |  |  |  |  |  |  |  |  |  |
| **AKI** |  | *Count* | *%* |  |  | *Missing (%)* |  | *Count* | *%* |  |  | *Missing (%)* |
| AKI |  | 527 | 0.8 |  |  | 0.0 |  | 337 | 0.5 |  |  | 0.0 |
| No AKI |  | 69,083 | 99.2 |  |  | 0.0 |  | 69,273 | 99.5 |  |  | 0.0 |
| **Gender** |  | *Male* | *Female* |  |  | *Missing (%)* |  | *Male* | *Female* |  |  | *Missing (%)* |
| AKI |  | 331 (62.8) | 196 (37.2) |  |  | 0.0 |  | 194 (57.6) | 143 (42.4) |  |  | 0.0 |
| No AKI |  | 37,500 (54.3) | 31,583 (45.7) |  |  | 0.0 |  | 37,637 (54.3) | 31,636 (45.7) |  |  | 0.0 |
| **Age at Exposure** |  | *<65* | *65-74* | *>=75* |  | *Missing (%)* |  | *<65* | *65-74* | *>=75* |  | *Missing (%)* |
| AKI |  | 191 (36.2) | 185 (35.1) | 151 (28.7) |  | 0.0 |  | 137 (40.7) | 102 (30.3) | 98 (29.1) |  | 0.0 |
| No AKI |  | 36,847 (53.3) | 19,168 (27.7) | 13,068 (18.9) |  | 0.0 |  | 36,302 (52.4) | 20,106 (29) | 12,865 (18.6) |  | 0.0 |
| **Diagnosis to Exposure** |  | *< 30 days* | *30-179* | *180-364* | *>= 365* | *Missing (%)* |  | *< 30 days* | *30-179* | *180-364* | *>= 365* | *Missing (%)* |
| AKI |  | 170 (32.3) | 61 (11.6) | 38 (7.2) | 258 (49) | 0.0 |  | 32 (9.5) | 124 (36.8) | 24 (7.1) | 157 (46.6) | 0.0 |
| No AKI |  | 26,074 (37.7) | 8,672 (12.6) | 4,003 (5.8) | 30,334 (43.9) | 0.0 |  | 7,134 (10.3) | 25,704 (37.1) | 5,580 (8.1) | 30,855 (44.5) | 0.0 |
| **# Medications** |  | *1 (%)* | *>= 2 (%)* |  |  | *Missing (%)* |  | *1 (%)* | *>= 2 (%)* |  |  | *Missing (%)* |
| AKI |  | 208 (39.5) | 319 (60.5) |  |  | 0.0 |  | 287 (85.2) | 50 (14.8) |  |  | 0.0 |
| No AKI |  | 34,877 (50.5) | 34,206 (49.5) |  |  | 0.0 |  | 59,642 (86.1) | 9,631 (13.9) |  |  | 0.0 |
| **# GP Consultations** |  | *<10* | *10-19* | *20-29* | *>=30* | *Missing (%)* |  | *<10* | *10-19* | *20-29* | *>=30* | *Missing (%)* |
| AKI |  | 130 (24.7) | 171 (32.4) | 118 (22.4) | 108 (20.5) | 0.0 |  | 72 (21.4) | 111 (32.9) | 78 (23.1) | 76 (22.6) | 0.0 |
| No AKI |  | 20,791 (30.1) | 24,665 (35.7) | 12,610 (18.3) | 11,017 (15.9) | 0.0 |  | 18,673 (27) | 25,574 (36.9) | 13,274 (19.2) | 11,752 (17) | 0.0 |
| **Systolic Blood Pressure** |  | < 120 | 120-139 | 140-159 | >=160 | *Missing (%)* |  | < 120 | 120-139 | 140-159 | >=160 | *Missing (%)* |
| AKI |  | 30 (5.9) | 127 (25.1) | 221 (43.8) | 127 (25.1) | 4.2 |  | 20 (6.2) | 136 (42) | 134 (41.4) | 34 (10.5) | 3.9 |
| No AKI |  | 1,843 (2.9) | 12,720 (19.8) | 30,560 (47.6) | 19,016 (29.6) | 7.2 |  | 5,112 (7.7) | 23,666 (35.5) | 28,440 (42.6) | 9,491 (14.2) | 3.7 |
| **Smoking** |  | *Yes (%)* | *No (%)* | *Ex (%)* |  | *Missing (%)* |  | *Yes (%)* | *No (%)* | *Ex (%)* |  | *Missing (%)* |
| AKI |  | 96 (18.3) | 220 (41.9) | 209 (39.8) |  | 0.4 |  | 70 (20.8) | 150 (44.6) | 116 (34.5) |  | 0.3 |
| No AKI |  | 12,026 (17.5) | 34,711 (50.5) | 22,056 (32.1) |  | 0.4 |  | 12,032 (17.4) | 35,098 (50.8) | 22,003 (31.8) |  | 0.2 |
| **GFR** |  | *>= 60* | *45-59* | *< 45* |  | *Missing (%)* |  | *>= 60* | *45-59* | *< 45* |  | *Missing (%)* |
| AKI |  | 343 (75.6) | 80 (17.6) | 31 (6.8) |  | 13.9 |  | 236 (77.9) | 50 (16.5) | 17 (5.6) |  | 10.1 |
| No AKI |  | 41,659 (86.2) | 5,686 (11.8) | 977 (2) |  | 30.1 |  | 37,505 (84.9) | 6,000 (13.6) | 648 (1.5) |  | 36.3 |
| **# Chronic Conditions** |  | *1 (%)* | *>= 2 (%)* |  |  | *Missing (%)* |  | *1 (%)* | *>= 2 (%)* |  |  | *Missing (%)* |
| AKI |  | 387 (73.4) | 140 (26.6) |  |  | 0.0 |  | 279 (82.8) | 58 (17.2) |  |  | 0.0 |
| No AKI |  | 56,521 (81.8) | 12,562 (18.2) |  |  | 0.0 |  | 61,312 (88.5) | 7,961 (11.5) |  |  | 0.0 |
| **Chronic Conditions** |  | *Count* | *%* |  |  |  |  | *Count* | *%* |  |  |  |
| Chronic Kidney Disease |  | 7,069 | 10.2 |  |  |  |  | 8,605 | 12.4 |  |  |  |
| Diabetes |  | 10,473 | 15.0 |  |  |  |  | 7,253 | 10.4 |  |  |  |
| Heart Failure |  | 0 | 0.0 |  |  |  |  | 0 | 0.0 |  |  |  |
| Hypertension |  | 54,636 | 78.5 |  |  |  |  | 48,959 | 70.3 |  |  |  |
| Ischaemic Heart Disease |  | 11,353 | 16.3 |  |  |  |  | 13,527 | 19.4 |  |  |  |

*Percentages exclude missing values, except for the “Missing” column which shows the percentage of patients with missing data.*

| **Table 2. Acute kidney injury rates (per 1,000 person-years) by covariables (non-missing)** | | | | | | | | | | |
| --- | --- | --- | --- | --- | --- | --- | --- | --- | --- | --- |
|  |  |  |  |  |  |  |  |  |  |  |
|  |  | **Exposed (n=39,586)** | | | |  | **Unexposed (n=39,586)** | | | |
|  |  |  |  |  |  |  |  |  |  |  |
| **Overall** |  | *Rate (95% CI)* |  |  |  |  | *Rate (95% CI)* |  |  |  |
|  |  | 2.3 (2.11-2.51) |  |  |  |  | 1.66 (1.49-1.84) |  |  |  |
| **Gender** |  | *Male* | *Female* |  |  |  | *Male* | *Female* |  |  |
|  |  | 2.64 (2.37-2.94) | 1.89 (1.64-2.17) |  |  |  | 1.75 (1.52-2.02) | 1.54 (1.31-1.82) |  |  |
| **Age at Exposure** |  | *<65* | *65-74* | *>=75* |  |  | *<65* | *65-74* | *>=75* |  |
|  |  | 1.53 (1.33-1.76) | 2.91 (2.52-3.36) | 3.71 (3.16-4.35) |  |  | 1.24 (1.05-1.47) | 1.72 (1.42-2.09) | 2.88 (2.36-3.51) |  |
| **Diagnosis to Exposure** |  | *< 30 days* | *30-179* | *180-364* | *>= 365* |  | *< 30 days* | *30-179* | *180-364* | *>= 365* |
|  |  | 2 (1.72-2.33) | 2.29 (1.78-2.94) | 3 (2.19-4.13) | 2.46 (2.18-2.78) |  | 1.79 (1.27-2.53) | 1.66 (1.4-1.98) | 1.47 (0.99-2.19) | 1.66 (1.42-1.94) |
| **# Medications** |  | *1* | *>= 2* |  |  |  | *1* | *>= 2* |  |  |
|  |  | 1.73 (1.51-1.99) | 2.92 (2.62-3.26) |  |  |  | 1.65 (1.47-1.86) | 1.66 (1.26-2.19) |  |  |
| **# GP Consultations** |  | *<10* | *10-19* | *20-29* | *>=30* |  | *<10* | *10-19* | *20-29* | *>=30* |
|  |  | 1.74 (1.47-2.07) | 2.06 (1.77-2.39) | 2.98 (2.49-3.57) | 3.41 (2.82-4.11) |  | 1.19 (0.94-1.49) | 1.44 (1.19-1.73) | 2.13 (1.71-2.66) | 2.62 (2.09-3.28) |
| **Systolic Blood Pressure** |  | < 120 | 120-139 | 140-159 | >=160 |  | < 120 | 120-139 | 140-159 | >=160 |
|  |  | 5.5 (3.85-7.87) | 3.12 (2.62-3.72) | 2.15 (1.89-2.46) | 1.95 (1.64-2.32) |  | 1.28 (0.83-1.99) | 1.87 (1.58-2.21) | 1.64 (1.39-1.94) | 1.29 (0.91-1.81) |
| **Smoking** |  | *Yes (%)* | *No (%)* | *Ex (%)* |  |  | *Yes (%)* | *No (%)* | *Ex (%)* |  |
|  |  | 2.44 (2-2.98) | 1.9 (1.67-2.17) | 2.87 (2.5-3.28) |  |  | 1.99 (1.57-2.52) | 1.45 (1.23-1.7) | 1.8 (1.5-2.16) |  |
| **GFR** |  | *>= 60* | *45-59* | *< 45* |  |  | *>= 60* | *45-59* | *< 45* |  |
|  |  | 2.41 (2.17-2.68) | 3.77 (3.03-4.69) | 9.06 (6.37-12.89) |  |  | 2 (1.76-2.27) | 2.58 (1.95-3.4) | 8.71 (5.33-14.21) |  |
| **# Chronic Conditions** |  | *1* | *>= 2* |  |  |  | *1* | *>= 2* |  |  |
|  |  | 2.09 (1.89-2.31) | 3.2 (2.71-3.77) |  |  |  | 1.54 (1.37-1.73) | 2.59 (2-3.35) |  |  |
| **Chronic Conditions** |  | *No* | *Yes* |  |  |  | *No* | *Yes* |  |  |
| Chronic Kidney Disease |  | 2.06 (1.88-2.27) | 4.4 (3.62-5.34) |  |  |  | 1.51 (1.34-1.7) | 2.75 (2.16-3.51) |  |  |
| Diabetes |  | 2.12 (1.92-2.33) | 3.34 (2.78-4.01) |  |  |  | 1.46 (1.29-1.64) | 3.62 (2.85-4.61) |  |  |
| Heart Failure |  | 2.3 (2.11-2.51) | no data |  |  |  | 1.66 (1.49-1.84) | no data |  |  |
| Hypertension |  | 3.89 (3.36-4.5) | 1.9 (1.71-2.11) |  |  |  | 2.2 (1.86-2.6) | 1.42 (1.24-1.63) |  |  |
| Ischaemic Heart Disease |  | 2.11 (1.92-2.33) | 3.3 (2.76-3.94) |  |  |  | 1.68 (1.49-1.89) | 1.57 (1.24-1.99) |  |  |

*Numbers in brackets are 95% confidence intervals*

| **Table 3. Cox Regression Models (n= 139,220)** | | |  |  |
| --- | --- | --- | --- | --- |
|  |  |  |  |  |
| **Model** | **Covariates** | **HR (AKI)** | **95% LCI** | **95% UCI** |
| Baseline^1^ | *Unexposed* | 1 |  |  |
|  | *Exposed* | 1.38 | 1.2 | 1.58 |
| Baseline + Sex | *Unexposed* | 1 |  |  |
|  | *Exposed* | 1.37 | 1.2 | 1.58 |
|  | *Male* | 1 |  |  |
|  | *Female* | 0.78 | 0.68 | 0.89 |
| Baseline + Age | *Unexposed* | 1 |  |  |
|  | *Exposed* | 1.37 | 1.19 | 1.57 |
|  | *< 65 years* | 1 |  |  |
|  | *65-74* | 1.7 | 1.45 | 1.99 |
|  | *>=75* | 2.44 | 2.07 | 2.88 |
| Baseline + Chronic_Time | *Unexposed* | 1 |  |  |
|  | *Exposed* | 1.43 | 1.23 | 1.65 |
|  | *< 30 days* | 1 |  |  |
|  | *30 - 179 days* | 1.14 | 0.92 | 1.41 |
|  | *180 - 364 days* | 1.23 | 0.92 | 1.64 |
|  | *>= 365 days* | 1.14 | 0.96 | 1.36 |
| Baseline + CKD | *Unexposed* | 1 |  |  |
|  | *Exposed* | 1.39 | 1.21 | 1.6 |
|  | *No CKD* | 1 |  |  |
|  | *CKD* | 2 | 1.69 | 2.37 |
| Baseline + DM | *Unexposed* | 1 |  |  |
|  | *Exposed* | 1.31 | 1.14 | 1.51 |
|  | *No DM* | 1 |  |  |
|  | *DM* | 1.86 | 1.57 | 2.19 |
| Baseline + HT | *Unexposed* | 1 |  |  |
|  | *Exposed* | 1.48 | 1.29 | 1.7 |
|  | *No HT* | 1 |  |  |
|  | *HT* | 0.55 | 0.48 | 0.63 |
| Baseline + IHD | *Unexposed* | 1 |  |  |
|  | *Exposed* | 1.4 | 1.22 | 1.6 |
|  | *No IHD* | 1 |  |  |
|  | *IHD* | 1.26 | 1.07 | 1.48 |
| Baseline + Medications | *Unexposed* | 1 |  |  |
|  | *Exposed* | 1.2 | 1.04 | 1.39 |
|  | *1* | 1 |  |  |
|  | *>= 2* | 1.47 | 1.27 | 1.7 |
| Baseline + GP Consultations | *Unexposed* | 1 |  |  |
|  | *Exposed* | 1.39 | 1.21 | 1.59 |
|  | *<10* | 1 |  |  |
|  | *10-19* | 1.21 | 1.01 | 1.45 |
|  | *20-29* | 1.79 | 1.47 | 2.18 |
|  | *>=30* | 2.16 | 1.76 | 2.63 |
| Baseline + SBP | *Unexposed* | 1 |  |  |
|  | *Exposed* | 1.52 | 1.32 | 1.75 |
|  | *<120* | 1 |  |  |
|  | *120-139* | 0.93 | 0.68 | 1.26 |
|  | *140-159* | 0.71 | 0.52 | 0.97 |
|  | *>=160* | 0.61 | 0.44 | 0.86 |
| Baseline + Smoking | *Unexposed* | 1 |  |  |
|  | *Exposed* | 1.38 | 1.2 | 1.58 |
|  | *No* | 1 |  |  |
|  | *Yes* | 1.33 | 1.1 | 1.59 |
|  | *Ex* | 1.41 | 1.21 | 1.63 |
| Basline + GFR | *Unexposed* | 1 |  |  |
|  | *Exposed* | 1.36 | 1.19 | 1.56 |
|  | *>=60* | 1 |  |  |
|  | *45-59* | 1.32 | 1.09 | 1.59 |
|  | *< 45* | 3.07 | 2.27 | 4.15 |
| Full Model | *Unexposed* | 1 |  |  |
|  | *Exposed* | 1.18 | 1 | 1.39 |
| Full Model (inc meds*exposure) | *1 (exposed)* | 0.97 | 0.79 | 1.19 |
|  | *>= 2 (exposed)* | 1.83 | 1.29 | 2.6 |

*^1^“Baseline” – hazard ratio prior to adjustment for covariable(s).*

| **Table 4. Cox Regression Models adjusted by Propensity Scores for Disease Severity (n = 139,220)** | | | | | |
| --- | --- | --- | --- | --- | --- |
|  |  |  |  |  |  |
|  |  |  |  |  |  |
| **Model** | **Covariates** | **HR (AKI)** | **95% LCI** | **95% UCI** |  |
| Baseline | *Unexposed* | 1 |  |  |  |
|  | *Exposed* | 1.38 | 1.2 | 1.58 |  |
| Baseline + P-Score (Full Model) | *Unexposed* | 1 |  |  |  |
|  | *Exposed* | 1.2 | 1.01 | 1.43 |  |
| Full Model (inc meds*exposure) | *1 (exposed)* | 1.03 | 0.83 | 1.28 |  |
|  | *>= 2 (exposed)* | 1.72 | 1.21 | 2.45 |  |
| ***Notes*** |  |  |  |  |  |
| *Variables in the propensity score model were: gender, age, time since first chronic condition, number of medications, number of GP consultations, chronic condition flags, systolic blood pressure, kidney function (GFR), and smoking status* | | | | |  |

| **Table 5. Prior-Event-Rate-Ratio (PERR) Analysis.** | | |  |
| --- | --- | --- | --- |
|  |  |  |  |
| *With left truncation at 3 years and excluding those with no days before exposure or AKI within 42 days; right truncation at 3 years (n=221,506)* | | | |
| **HR (prior)** | **HR (post)** | **PERR** |  |
| 1.26 (0.96-1.66) | 1.59 (1.35-1.86) | 1.26 (0.85-1.67)* |  |
|  |  |  |  |
| ** Bootstrapped confidence interval; reps=100* | | |  |
