# Supplementary material for: Estimating the risk of acute kidney injury associated with use of diuretics and renin angiotensin aldosterone system inhibitors: A population based cohort study using the clinical practice research datalink
Source: BMC Nephrol. 2019 Dec 30;20:481. doi: 10.1186/s12882-019-1633-2 (PMC6937998; doi:10.1186/s12882-019-1633-2)
Supplement: Supplementary file 3 — Additional file 3. Analyses limited to those patients eligible for linkage to Hospital Episode Statistics and quintiles of the Index of Multiple Deprivation. A sensitivity analysis. [file 12882_2019_1633_MOESM3_ESM.docx]

| **Table 1. Covariable information for non-missing data, by exposure (RAAS blockers / diuretics) and outcome (acute kidney injury)** | | | | | | | | | | | | |
| --- | --- | --- | --- | --- | --- | --- | --- | --- | --- | --- | --- | --- |
|  |  |  |  |  |  |  |  |  |  |  |  |  |
|  |  | **Exposed (n=41,422)** | | | | |  | **Unexposed (n=42,774)** | | | | |
|  |  |  |  |  |  |  |  |  |  |  |  |  |
| **AKI** |  | *Count* | *%* |  |  | *Missing (%)* |  | *Count* | *%* |  |  | *Missing (%)* |
| AKI |  | 424 | 1.0 |  |  | 0.0 |  | 265 | 0.6 |  |  | 0.0 |
| No AKI |  | 40,998 | 99.0 |  |  | 0.0 |  | 42,509 | 99.4 |  |  | 0.0 |
| **Gender** |  | *Male* | *Female* |  |  | *Missing (%)* |  | *Male* | *Female* |  |  | *Missing (%)* |
| AKI |  | 258 (60.8) | 166 (39.2) |  |  | 0.0 |  | 153 (57.7) | 112 (42.3) |  |  | 0.0 |
| No AKI |  | 22,346 (54.5) | 18,652 (45.5) |  |  | 0.0 |  | 23,051 (54.2) | 19,458 (45.8) |  |  | 0.0  **Additional File 3.**  **Sensitivity analysis: Analyses limited to those patients eligible for linkage to Hospital Episode Statistics and quintiles of the Index of Multiple Deprivation** |
| **Age at Exposure** |  | *<65* | *65-74* | *>=75* |  | *Missing (%)* |  | *<65* | *65-74* | *>=75* |  | *Missing (%)* |
| AKI |  | 194 (45.8) | 127 (30) | 103 (24.3) |  | 0.0 |  | 110 (41.5) | 93 (35.1) | 62 (23.4) |  | 0.0 |
| No AKI |  | 21,586 (52.7) | 11,290 (27.5) | 8,122 (19.8) |  | 0.0 |  | 22,157 (52.1) | 12,307 (29) | 8,045 (18.9) |  | 0.0 |
| **Diagnosis to Exposure** |  | *< 30 days* | *30-179* | *180-364* | *>= 365* | *Missing (%)* |  | *< 30 days* | *30-179* | *180-364* | *>= 365* | *Missing (%)* |
| AKI |  | 149 (35.1) | 63 (14.9) | 32 (7.5) | 180 (42.5) | 0.0 |  | 28 (10.6) | 88 (33.2) | 28 (10.6) | 121 (45.7) | 0.0 |
| No AKI |  | 15,187 (37) | 5,181 (12.6) | 2,347 (5.7) | 18,283 (44.6) | 0.0 |  | 4,424 (10.4) | 15,804 (37.2) | 3,505 (8.2) | 18,776 (44.2) | 0.0 |
| **# Medications** |  | *1 (%)* | *>= 2 (%)* |  |  | *Missing (%)* |  | *1 (%)* | *>= 2 (%)* |  |  | *Missing (%)* |
| AKI |  | 199 (46.9) | 225 (53.1) |  |  | 0.0 |  | 223 (84.2) | 42 (15.8) |  |  | 0.0 |
| No AKI |  | 20,256 (49.4) | 20,742 (50.6) |  |  | 0.0 |  | 36,722 (86.4) | 5,787 (13.6) |  |  | 0.0 |
| **# GP Consultations** |  | *<10* | *10-19* | *20-29* | *>=30* | *Missing (%)* |  | *<10* | *10-19* | *20-29* | *>=30* | *Missing (%)* |
| AKI |  | 117 (27.6) | 148 (34.9) | 87 (20.5) | 72 (17) | 0.0 |  | 59 (22.3) | 99 (37.4) | 61 (23) | 46 (17.4) | 0.0 |
| No AKI |  | 11,899 (29) | 14,849 (36.2) | 7,697 (18.8) | 6,553 (16) | 0.0 |  | 11,115 (26.1) | 15,994 (37.6) | 8,320 (19.6) | 7,080 (16.7) | 0.0 |
| **Systolic Blood Pressure** |  | < 120 | 120-139 | 140-159 | >=160 | *Missing (%)* |  | < 120 | 120-139 | 140-159 | >=160 | *Missing (%)* |
| AKI |  | 17 (4.3) | 99 (24.9) | 171 (43.1) | 110 (27.7) | 6.4 |  | 16 (6.1) | 100 (38.2) | 116 (44.3) | 30 (11.5) | 1.1 |
| No AKI |  | 1,179 (3.1) | 8,029 (21.1) | 18,004 (47.3) | 10,821 (28.5) | 7.2 |  | 2,979 (7.3) | 14,403 (35.1) | 17,772 (43.3) | 5,873 (14.3) | 3.5 |
| **Smoking** |  | *Yes (%)* | *No (%)* | *Ex (%)* |  | *Missing (%)* |  | *Yes (%)* | *No (%)* | *Ex (%)* |  | *Missing (%)* |
| AKI |  | 78 (18.5) | 189 (44.9) | 154 (36.6) |  | 0.7 |  | 54 (20.4) | 117 (44.2) | 94 (35.5) |  | 0.0 |
| No AKI |  | 6,796 (16.7) | 20,614 (50.5) | 13,400 (32.8) |  | 0.5 |  | 6,905 (16.3) | 21,861 (51.5) | 13,660 (32.2) |  | 0.2 |
| **GFR** |  | *>= 60* | *45-59* | *< 45* |  | *Missing (%)* |  | *>= 60* | *45-59* | *< 45* |  | *Missing (%)* |
| AKI |  | 309 (82.2) | 48 (12.8) | 19 (5.1) |  | 11.3 |  | 209 (85.7) | 23 (9.4) | 12 (4.9) |  | 7.9 |
| No AKI |  | 23,495 (86.5) | 3,087 (11.4) | 577 (2.1) |  | 33.8 |  | 22,081 (87.3) | 2,870 (11.3) | 337 (1.3) |  | 40.5 |
| **# Chronic Conditions** |  | *1 (%)* | *>= 2 (%)* |  |  | *Missing (%)* |  | *1 (%)* | *>= 2 (%)* |  |  | *Missing (%)* |
| AKI |  | 316 (74.5) | 108 (25.5) |  |  | 0.0 |  | 220 (83) | 45 (17) |  |  | 0.0 |
| No AKI |  | 32,926 (80.3) | 8,072 (19.7) |  |  | 0.0 |  | 37,775 (88.9) | 4,734 (11.1) |  |  | 0.0 |
| **Chronic Conditions** |  | *Count* | *%* |  |  |  |  | *Count* | *%* |  |  |  |
| Chronic Kidney Disease |  | 4,320 | 10.4 |  |  |  |  | 4,877 | 11.4 |  |  |  |
| Diabetes |  | 6,409 | 15.5 |  |  |  |  | 4,391 | 10.3 |  |  |  |
| Heart Failure |  | 1,222 | 3.0 |  |  |  |  | 155 | 0.4 |  |  |  |
| Hypertension |  | 31,973 | 77.2 |  |  |  |  | 30,827 | 72.1 |  |  |  |
| Ischaemic Heart Disease |  | 6,579 | 15.9 |  |  |  |  | 7,702 | 18.0 |  |  |  |
|  |  |  |  |  |  |  |  |  |  |  |  |  |

*Percentages exclude missing values, except for the “Missing” column which shows the percentage of patients with missing data.*

| **Table 2. Acute kidney injury rates (per 1,000 person-years) by covariables (non-missing)** | | | | | | | | | | |
| --- | --- | --- | --- | --- | --- | --- | --- | --- | --- | --- |
|  |  |  |  |  |  |  |  |  |  |  |
|  |  | **Exposed (n=41,422)** | | | |  | **Unexposed (n=42,774)** | | | |
|  |  |  |  |  |  |  |  |  |  |  |
| **Overall** |  | *Rate (95% CI)* |  |  |  |  | *Rate (95% CI)* |  |  |  |
|  |  | 3.25 (2.95-3.57) |  |  |  |  | 2.24 (1.98-2.52) |  |  |  |
| **Gender** |  | *Male* | *Female* |  |  |  | *Male* | *Female* |  |  |
|  |  | 3.61 (3.19-4.08) | 2.81 (2.41-3.27) |  |  |  | 2.38 (2.03-2.79) | 2.06 (1.71-2.48) |  |  |
| **Age at Exposure** |  | *<65* | *65-74* | *>=75* |  |  | *<65* | *65-74* | *>=75* |  |
|  |  | 2.75 (2.39-3.17) | 3.52 (2.96-4.19) | 4.27 (3.52-5.18) |  |  | 1.71 (1.42-2.07) | 2.71 (2.21-3.33) | 3.07 (2.39-3.93) |  |
| **Diagnosis to Exposure** |  | *< 30 days* | *30-179* | *180-364* | *>= 365* |  | *< 30 days* | *30-179* | *180-364* | *>= 365* |
|  |  | 3.16 (2.69-3.71) | 4.12 (3.22-5.27) | 4.39 (3.11-6.21) | 2.95 (2.55-3.42) |  | 2.73 (1.88-3.95) | 2.02 (1.64-2.49) | 2.92 (2.01-4.22) | 2.19 (1.83-2.62) |
| **# Medications** |  | *1* | *>= 2* |  |  |  | *1* | *>= 2* |  |  |
|  |  | 2.97 (2.58-3.41) | 3.54 (3.11-4.03) |  |  |  | 2.2 (1.93-2.51) | 2.46 (1.82-3.33) |  |  |
| **# GP Consultations** |  | *<10* | *10-19* | *20-29* | *>=30* |  | *<10* | *10-19* | *20-29* | *>=30* |
|  |  | 2.86 (2.39-3.43) | 3.07 (2.61-3.61) | 3.71 (3-4.57) | 3.99 (3.17-5.03) |  | 1.72 (1.33-2.22) | 2.15 (1.77-2.63) | 2.79 (2.17-3.59) | 2.79 (2.09-3.73) |
| **Systolic Blood Pressure** |  | < 120 | 120-139 | 140-159 | >=160 |  | < 120 | 120-139 | 140-159 | >=160 |
|  |  | 5.26 (3.27-8.46) | 4.06 (3.33-4.94) | 2.95 (2.54-3.43) | 3.05 (2.53-3.67) |  | 1.87 (1.15-3.06) | 2.38 (1.96-2.89) | 2.39 (1.99-2.86) | 1.93 (1.34-2.78) |
| **Smoking** |  | *Yes (%)* | *No (%)* | *Ex (%)* |  |  | *Yes (%)* | *No (%)* | *Ex (%)* |  |
|  |  | 3.66 (2.93-4.57) | 2.86 (2.48-3.3) | 3.62 (3.09-4.24) |  |  | 2.87 (2.2-3.75) | 1.9 (1.58-2.28) | 2.48 (2.02-3.03) |  |
| **GFR** |  | *>= 60* | *45-59* | *< 45* |  |  | *>= 60* | *45-59* | *< 45* |  |
|  |  | 3.89 (3.48-4.35) | 4.3 (3.24-5.71) | 9.61 (6.13-15.06) |  |  | 3.07 (2.68-3.52) | 2.57 (1.71-3.87) | 11.03 (6.11-19.92) |  |
| **# Chronic Conditions** |  | *1* | *>= 2* |  |  |  | *1* | *>= 2* |  |  |
|  |  | 3.04 (2.73-3.4) | 4.02 (3.33-4.86) |  |  |  | 2.08 (1.82-2.37) | 3.54 (2.64-4.74) |  |  |
| **Chronic Conditions** |  | *No* | *Yes* |  |  |  | *No* | *Yes* |  |  |
| Chronic Kidney Disease |  | 3.16 (2.85-3.5) | 3.99 (3.06-5.2) |  |  |  | 2.16 (1.9-2.46) | 2.85 (2.06-3.93) |  |  |
| Diabetes |  | 2.93 (2.62-3.26) | 4.99 (4.1-6.06) |  |  |  | 1.95 (1.71-2.24) | 5.1 (3.91-6.66) |  |  |
| Heart Failure |  | 3.03 (2.74-3.35) | 12.66 (9.17-17.47) |  |  |  | 2.23 (1.98-2.52) | 3.24 (0.46-22.98) |  |  |
| Hypertension |  | 5.03 (4.25-5.94) | 2.78 (2.47-3.12) |  |  |  | 2.97 (2.44-3.62) | 1.94 (1.67-2.26) |  |  |
| Ischaemic Heart Disease |  | 3.17 (2.85-3.52) | 3.68 (2.93-4.62) |  |  |  | 2.18 (1.9-2.5) | 2.47 (1.91-3.19) |  |  |
|  |  |  |  |  |  |  |  |  |  |  |

*Numbers in brackets are 95% confidence intervals*

| **Table 3. Cox Regression Models (n= 84,196)** | | |  |  |
| --- | --- | --- | --- | --- |
|  |  |  |  |  |
| **Model** | **Covariates** | **HR (AKI)** | **95% LCI** | **95% UCI** |
| Baseline^1^ | *Unexposed* | 1 |  |  |
|  | *Exposed* | 1.43 | 1.23 | 1.67 |
| Baseline + Sex | *Unexposed* | 1 |  |  |
|  | *Exposed* | 1.43 | 1.23 | 1.67 |
|  | *Male* | 1 |  |  |
|  | *Female* | 0.81 | 0.7 | 0.95 |
| Baseline + Age | *Unexposed* | 1 |  |  |
|  | *Exposed* | 1.43 | 1.23 | 1.67 |
|  | *< 65 years* | 1 |  |  |
|  | *65-74* | 1.4 | 1.18 | 1.67 |
|  | *>=75* | 1.67 | 1.39 | 2.02 |
| Baseline + Chronic_Time | *Unexposed* | 1 |  |  |
|  | *Exposed* | 1.46 | 1.23 | 1.72 |
|  | *< 30 days* | 1 |  |  |
|  | *30 - 179 days* | 1.03 | 0.81 | 1.3 |
|  | *180 - 364 days* | 1.32 | 0.98 | 1.78 |
|  | *>= 365 days* | 0.91 | 0.76 | 1.11 |
| Baseline + CKD | *Unexposed* | 1 |  |  |
|  | *Exposed* | 1.44 | 1.23 | 1.67 |
|  | *No CKD* | 1 |  |  |
|  | *CKD* | 1.28 | 1.03 | 1.6 |
| Baseline + DM | *Unexposed* | 1 |  |  |
|  | *Exposed* | 1.35 | 1.16 | 1.58 |
|  | *No DM* | 1 |  |  |
|  | *DM* | 1.97 | 1.65 | 2.36 |
| Baseline + HF | *Unexposed* | 1 |  |  |
|  | *Exposed* | 1.35 | 1.16 | 1.58 |
|  | *No HF* | 1 |  |  |
|  | *HF* | 4.21 | 3.02 | 5.86 |
| Baseline + HT | *Unexposed* | 1 |  |  |
|  | *Exposed* | 1.51 | 1.29 | 1.76 |
|  | *No HT* | 1 |  |  |
|  | *HT* | 0.59 | 0.5 | 0.69 |
| Baseline + IHD | *Unexposed* | 1 |  |  |
|  | *Exposed* | 1.44 | 1.24 | 1.68 |
|  | *No IHD* | 1 |  |  |
|  | *IHD* | 1.14 | 0.94 | 1.38 |
| Baseline + Medications | *Unexposed* | 1 |  |  |
|  | *Exposed* | 1.35 | 1.15 | 1.59 |
|  | *1* | 1 |  |  |
|  | *>= 2* | 1.18 | 1 | 1.39 |
| Baseline + GP Consultations | *Unexposed* | 1 |  |  |
|  | *Exposed* | 1.44 | 1.23 | 1.68 |
|  | *<10* | 1 |  |  |
|  | *10-19* | 1.15 | 0.95 | 1.39 |
|  | *20-29* | 1.45 | 1.16 | 1.8 |
|  | *>=30* | 1.54 | 1.22 | 1.95 |
| Baseline + SBP | *Unexposed* | 1 |  |  |
|  | *Exposed* | 1.51 | 1.29 | 1.77 |
|  | *<120* | 1 |  |  |
|  | *120-139* | 1.01 | 0.7 | 1.46 |
|  | *140-159* | 0.84 | 0.59 | 1.21 |
|  | *>=160* | 0.79 | 0.54 | 1.15 |
| Baseline + Smoking | *Unexposed* | 1 |  |  |
|  | *Exposed* | 1.43 | 1.23 | 1.67 |
|  | *No* | 1 |  |  |
|  | *Yes* | 1.38 | 1.13 | 1.7 |
|  | *Ex* | 1.28 | 1.09 | 1.52 |
| Basline + GFR | *Unexposed* | 1 |  |  |
|  | *Exposed* | 1.42 | 1.22 | 1.66 |
|  | *>=60* | 1 |  |  |
|  | *45-59* | 1 | 0.78 | 1.28 |
|  | *< 45* | 2.51 | 1.74 | 3.62 |
| Full Model | *Unexposed* | 1 |  |  |
|  | *Exposed* | 1.16 | 0.96 | 1.41 |
| Full Model (inc meds*exposure) | *1 (exposed)* | 1.13 | 0.91 | 1.41 |
|  | *>= 2 (exposed)* | 1.11 | 0.75 | 1.63 |

*^1^“Baseline” – hazard ratio prior to adjustment for covariable(s).*

| **Table 4. Cox Regression Models adjusted by Propensity Scores for Disease Severity^1^ (n = 84,196)** | | | | | |
| --- | --- | --- | --- | --- | --- |
|  |  |  |  |  |  |
|  |  |  |  |  |  |
| **Model** | **Covariates** | **HR (AKI)** | **95% LCI** | **95% UCI** |  |
| Baseline | *Unexposed* | 1 |  |  |  |
|  | *Exposed* | 1.43 | 1.23 | 1.67 |  |
| Baseline + P-Score (Full Model) | *Unexposed* | 1 |  |  |  |
|  | *Exposed* | 1.17 | 0.96 | 1.43 |  |
| Full Model (inc meds*exposure) | *1 (exposed)* | 1.11 | 0.88 | 1.4 |  |
|  | *>= 2 (exposed)* | 1.2 | 0.82 | 1.77 |  |
| ***Notes*** |  |  |  |  |  |
| *Variables in the propensity score model were: gender, age, time since first chronic condition, number of medications, number of GP consultations, chronic condition flags, systolic blood pressure, kidney function (GFR), and smoking status* | | | | |  |

| **Table 5. Prior-Event-Rate-Ratio (PERR) Analysis.** | | |  |
| --- | --- | --- | --- |
|  |  |  |  |
| *With left truncation at 3 years and excluding those with no days before exposure or AKI within 42 days and excluding those not eligible for linkage to HES and ONS; right truncation at 3 years (n=148,148)* | | | |
| **HR (prior)** | **HR (post)** | **PERR** |  |
| 1.45 (1.08-1.95) | 1.55 (1.30-1.84) | 1.06 (0.71-1.41)* |  |
|  |  |  |  |
| ** Bootstrapped confidence interval; reps=100* | | |  |
