# Supplementary material for: Estimating the risk of acute kidney injury associated with use of diuretics and renin angiotensin aldosterone system inhibitors: A population based cohort study using the clinical practice research datalink
Source: BMC Nephrol. 2019 Dec 30;20:481. doi: 10.1186/s12882-019-1633-2 (PMC6937998; doi:10.1186/s12882-019-1633-2)
Supplement: Supplementary file 4 — Additional file 4. Sensitivity analysis comparing individuals prescribed either RAAS inhibitors or diuretics alone, with those receiving both classes of medication. [file 12882_2019_1633_MOESM4_ESM.docx]

**Additional File 4.**

**Sensitivity analysis: Sensitivity analysis comparing individuals prescribed either RAAS inhibitors or diuretics alone, with those receiving both classes of medication.**

| **Table 1. Covariate information for non-missing data, by exposure (RAAS blockers) and outcome (AKI)** | | | | | | | | | | | | |  |  |  | |
| --- | --- | --- | --- | --- | --- | --- | --- | --- | --- | --- | --- | --- | --- | --- | --- | --- |
|  |  |  | |  |  | |  |  |  |  | |  |  |  |  | |
|  |  | **Exposed (n=17,517)** | | | | | | |  | **Unexposed (n=17,517)** | | | | | | |
|  |  |  |  | |  |  | |  |  |  |  | |  |  | |  |
| **AKI** |  | *Count* | *%* | |  |  | | *Missing (%)* |  | *Count* | *%* | |  |  | | *Missing (%)* |
| AKI |  | 232 | 1.3 | |  |  | | 0.0 |  | 86 | 0.5 | |  |  | | 0.0 |
| No AKI |  | 17,285 | 98.7 | |  |  | | 0.0 |  | 17,431 | 99.5 | |  |  | | 0.0 |
| **Gender** |  | *Male* | *Female* | |  |  | | *Missing (%)* |  | *Male* | *Female* | |  |  | | *Missing (%)* |
| AKI |  | 134 (57.8) | 98 (42.2) | |  |  | | 0.0 |  | 53 (61.6) | 33 (38.4) | |  |  | | 0.0 |
| No AKI |  | 8,991 (52) | 8,294 (48) | |  |  | | 0.0 |  | 9,072 (52) | 8,359 (48) | |  |  | | 0.0 |
| **Age at Exposure** |  | *<65* | *65-74* | | *>=75* |  | | *Missing (%)* |  | *<65* | *65-74* | | *>=75* |  | | *Missing (%)* |
| AKI |  | 75 (32.3) | 75 (32.3) | | 82 (35.3) |  | | 0.0 |  | 21 (24.4) | 29 (33.7) | | 36 (41.9) |  | | 0.0 |
| No AKI |  | 7,656 (44.3) | 5,398 (31.2) | | 4,231 (24.5) |  | | 0.0 |  | 7,611 (43.7) | 5,644 (32.4) | | 4,176 (24) |  | | 0.0 |
| **Diagnosis to Exposure** |  | *< 30 days* | *30-179* | | *180-364* | *>= 365* | | *Missing (%)* |  | *< 30 days* | *30-179* | | *180-364* | *>= 365* | | *Missing (%)* |
| AKI |  | 67 (28.9) | 40 (17.2) | | 16 (6.9) | 109 (47) | | 0.0 |  | 9 (10.5) | 26 (30.2) | | 11 (12.8) | 40 (46.5) | | 0.0 |
| No AKI |  | 6,335 (36.7) | 2,455 (14.2) | | 969 (5.6) | 7,526 (43.5) | | 0.0 |  | 1,791 (10.3) | 6,440 (36.9) | | 1,464 (8.4) | 7,736 (44.4) | | 0.0 |
| **# Anti-hypertensives** |  | *1 (%)* | *>= 2 (%)* | |  |  | | *Missing (%)* |  | *1 (%)* | *>= 2 (%)* | |  |  | | *Missing (%)* |
| AKI |  | 71 (30.6) | 161 (69.4) | |  |  | | 0.0 |  | 75 (87.2) | 11 (12.8) | |  |  | | 0.0 |
| No AKI |  | 7,200 (41.7) | 10,085 (58.3) | |  |  | | 0.0 |  | 14,841 (85.1) | 2,590 (14.9) | |  |  | | 0.0 |
| **# GP Consultations** |  | *<10* | *10-19* | | *20-29* | *>=30* | | *Missing (%)* |  | *<10* | *10-19* | | *20-29* | *>=30* | | *Missing (%)* |
| AKI |  | 51 (22) | 80 (34.5) | | 50 (21.6) | 51 (22) | | 0.0 |  | 15 (17.4) | 29 (33.7) | | 22 (25.6) | 20 (23.3) | | 0.0 |
| No AKI |  | 5,340 (30.9) | 6,071 (35.1) | | 3,104 (18) | 2,770 (16) | | 0.0 |  | 4,798 (27.5) | 6,526 (37.4) | | 3,243 (18.6) | 2,864 (16.4) | | 0.0 |
| **Systolic Blood Pressure** |  | < 120 | 120-139 | | 140-159 | >=160 | | *Missing (%)* |  | < 120 | 120-139 | | 140-159 | >=160 | | *Missing (%)* |
| AKI |  | 12 (5.5) | 46 (21.2) | | 82 (37.8) | 77 (35.5) | | 6.5 |  | 7 (8.3) | 28 (33.3) | | 38 (45.2) | 11 (13.1) | | 2.3 |
| No AKI |  | 455 (2.8) | 2,767 (17.3) | | 6,904 (43.2) | 5,844 (36.6) | | 7.6 |  | 1,286 (7.6) | 5,946 (35.3) | | 7,128 (42.4) | 2,470 (14.7) | | 3.4 |
| **Smoking** |  | *Yes (%)* | *No (%)* | | *Ex (%)* |  | | *Missing (%)* |  | *Yes (%)* | *No (%)* | | *Ex (%)* |  | | *Missing (%)* |
| AKI |  | 48 (21.1) | 93 (41) | | 86 (37.9) |  | | 2.2 |  | 15 (17.4) | 36 (41.9) | | 35 (40.7) |  | | 0.0 |
| No AKI |  | 3,020 (17.6) | 8,410 (49) | | 5,740 (33.4) |  | | 0.7 |  | 2,819 (16.2) | 8,795 (50.5) | | 5,787 (33.3) |  | | 0.2 |
| **GFR** |  | *>= 60* | *45-59* | | *< 45* |  | | *Missing (%)* |  | *>= 60* | *45-59* | | *< 45* |  | | *Missing (%)* |
| AKI |  | 150 (77.3) | 35 (18) | | 9 (4.6) |  | | 16.4 |  | 61 (77.2) | 13 (16.5) | | 5 (6.3) |  | | 8.1 |
| No AKI |  | 10,110 (83.5) | 1,662 (13.7) | | 335 (2.8) |  | | 30.0 |  | 8,701 (82.5) | 1,646 (15.6) | | 203 (1.9) |  | | 39.5 |
| **# Chronic Conditions** |  | *1 (%)* | *>= 2 (%)* | |  |  | | *Missing (%)* |  | *1 (%)* | *>= 2 (%)* | |  |  | | *Missing (%)* |
| AKI |  | 159 (68.5) | 73 (31.5) | |  |  | | 0.0 |  | 72 (83.7) | 14 (16.3) | |  |  | | 0.0 |
| No AKI |  | 13,746 (79.5) | 3,539 (20.5) | |  |  | | 0.0 |  | 15,313 (87.8) | 2,118 (12.2) | |  |  | | 0.0 |
| **Chronic Conditions** |  | *Count* | *%* | |  |  | |  |  | *Count* | *%* | |  |  | |  |
| Chronic Kidney Disease |  | 1,736 | 9.9 | |  |  | |  |  | 2,350 | 13.4 | |  |  | |  |
| Diabetes |  | 1,832 | 10.5 | |  |  | |  |  | 1,639 | 9.4 | |  |  | |  |
| Heart Failure |  | 1,296 | 7.4 | |  |  | |  |  | 63 | 0.4 | |  |  | |  |
| Hypertension |  | 13,870 | 79.2 | |  |  | |  |  | 12,104 | 69.1 | |  |  | |  |
| Ischaemic Heart Disease |  | 2,911 | 16.6 | |  |  | |  |  | 3,709 | 21.2 | |  |  | |  |
| **Other Medications** |  | *Count* | *%* | |  |  | |  |  | *Count* | *%* | |  |  | |  |
| NSAIDS |  | 94 | 0.5 | |  |  | |  |  | 268 | 1.5 | |  |  | |  |

| **Table 2. AKI Rates (per 1,000 person-years) by covariates (non-missing)** | | | | | | | | | | |
| --- | --- | --- | --- | --- | --- | --- | --- | --- | --- | --- |
|  |  | **Exposed (n=17,517)** | | | |  | **Unexposed (n=17,517)** | | | |
|  |  |  |  |  |  |  |  |  |  |  |
| **Overall** |  | *Rate (95% CI)* |  |  |  |  | *Rate (95% CI)* |  |  |  |
|  |  | 3.36 (2.95-3.82) |  |  |  |  | 1.5 (1.21-1.85) |  |  |  |
| **Gender** |  | *Male* | *Female* |  |  |  | *Male* | *Female* |  |  |
|  |  | 3.74 (3.16-4.43) | 2.95 (2.42-3.6) |  |  |  | 1.77 (1.35-2.32) | 1.2 (0.85-1.68) |  |  |
| **Age at Exposure** |  | *<65* | *65-74* | *>=75* |  |  | *<65* | *65-74* | *>=75* |  |
|  |  | 2.35 (1.87-2.94) | 3.5 (2.79-4.38) | 5.24 (4.22-6.5) |  |  | 0.79 (0.51-1.21) | 1.56 (1.08-2.24) | 2.94 (2.12-4.08) |  |
| **Diagnosis to Exposure** |  | *< 30 days* | *30-179* | *180-364* | *>= 365* |  | *< 30 days* | *30-179* | *180-364* | *>= 365* |
|  |  | 2.73 (2.15-3.47) | 4.53 (3.32-6.17) | 4.08 (2.5-6.66) | 3.43 (2.84-4.14) |  | 1.78 (0.93-3.42) | 1.24 (0.84-1.81) | 2.36 (1.31-4.26) | 1.5 (1.1-2.04) |
| **# Medications** |  | *1* | *>= 2* |  |  |  | *1* | *>= 2* |  |  |
|  |  | 2.25 (1.78-2.84) | 4.3 (3.68-5.01) |  |  |  | 1.54 (1.23-1.93) | 1.25 (0.69-2.25) |  |  |
| **# GP Consultations** |  | *<10* | *10-19* | *20-29* | *>=30* |  | *<10* | *10-19* | *20-29* | *>=30* |
|  |  | 2.25 (1.71-2.96) | 3.29 (2.64-4.09) | 4.12 (3.12-5.43) | 5.15 (3.91-6.77) |  | 0.86 (0.52-1.42) | 1.33 (0.92-1.91) | 2.18 (1.43-3.31) | 2.5 (1.61-3.87) |
| **Systolic Blood Pressure** |  | < 120 | 120-139 | 140-159 | >=160 |  | < 120 | 120-139 | 140-159 | >=160 |
|  |  | 8.13 (4.62-14.31) | 4.5 (3.37-6) | 2.85 (2.3-3.54) | 3.23 (2.58-4.03) |  | 1.66 (0.79-3.49) | 1.36 (0.94-1.98) | 1.62 (1.18-2.23) | 1.48 (0.82-2.67) |
| **Smoking** |  | *Yes (%)* | *No (%)* | *Ex (%)* |  |  | *Yes (%)* | *No (%)* | *Ex (%)* |  |
|  |  | 3.98 (3-5.29) | 2.76 (2.25-3.38) | 3.76 (3.05-4.65) |  |  | 1.65 (0.99-2.73) | 1.23 (0.89-1.71) | 1.84 (1.32-2.56) |  |
| **GFR** |  | *>= 60* | *45-59* | *< 45* |  |  | *>= 60* | *45-59* | *< 45* |  |
|  |  | 3.6 (3.07-4.22) | 4.9 (3.52-6.82) | 6.77 (3.52-13.01) |  |  | 1.91 (1.49-2.46) | 2.15 (1.25-3.71) | 7.83 (3.26-18.82) |  |
| **# Chronic Conditions** |  | *1* | *>= 2* |  |  |  | *1* | *>= 2* |  |  |
|  |  | 2.9 (2.48-3.39) | 5.13 (4.08-6.46) |  |  |  | 1.42 (1.13-1.79) | 2.09 (1.24-3.53) |  |  |
| **Chronic Conditions** |  | *No* | *Yes* |  |  |  | *No* | *Yes* |  |  |
| Chronic Kidney Disease |  | 3.2 (2.79-3.68) | 4.75 (3.37-6.67) |  |  |  | 1.35 (1.07-1.71) | 2.52 (1.59-3.99) |  |  |
| Diabetes |  | 2.97 (2.57-3.44) | 6.53 (4.93-8.64) |  |  |  | 1.31 (1.03-1.66) | 3.55 (2.21-5.71) |  |  |
| Heart Failure |  | 3.02 (2.63-3.47) | 9.83 (7.02-13.75) |  |  |  | 1.48 (1.2-1.83) | 5.88 (0.83-41.73) |  |  |
| Hypertension |  | 6.62 (5.33-8.24) | 2.66 (2.27-3.12) |  |  |  | 2.17 (1.59-2.97) | 1.19 (0.89-1.58) |  |  |
| Ischaemic Heart Disease |  | 3.02 (2.61-3.5) | 5.26 (4.04-6.86) |  |  |  | 1.49 (1.17-1.89) | 1.52 (0.98-2.36) |  |  |
| **Other Medications** |  | *No* | *Yes* |  |  |  | *No* | *Yes* |  |  |
| NSAIDS |  | 3.35 (2.94-3.81) | 5.65 (1.41-22.58) |  |  |  | 1.52 (1.23-1.88) | 0 (0-0) |  |  |

| **Table 3. Cox Regression Models (n= 35,034)** | | | |  |
| --- | --- | --- | --- | --- |
|  |  |  |  | |
| **Model** | **Covariates** | **HR (AKI)** | **95% CI** | |
| Baseline | *Unexposed* | 1 |  |  |
|  | *Exposed* | 2.22 | 1.73 | 2.84 |
| Baseline + Sex | *Unexposed* | 1 |  |  |
|  | *Exposed* | 2.22 | 1.73 | 2.85 |
|  | *Male* | 1 |  |  |
|  | *Female* | 0.76 | 0.61 | 0.95 |
| Baseline + Age | *Unexposed* | 1 |  |  |
|  | *Exposed* | 2.19 | 1.71 | 2.81 |
|  | *< 65 years* | 1 |  |  |
|  | *65-74* | 1.62 | 1.23 | 2.14 |
|  | *>=75* | 2.63 | 2.01 | 3.45 |
| Baseline + Chronic_Time | *Unexposed* | 1 |  |  |
|  | *Exposed* | 2.4 | 1.85 | 3.11 |
|  | *< 30 days* | 1 |  |  |
|  | *30 - 179 days* | 1.33 | 0.94 | 1.88 |
|  | *180 - 364 days* | 1.6 | 1.03 | 2.49 |
|  | *>= 365 days* | 1.19 | 0.9 | 1.58 |
| Baseline + CKD | *Unexposed* | 1 |  |  |
|  | *Exposed* | 2.25 | 1.75 | 2.88 |
|  | *No CKD* | 1 |  |  |
|  | *CKD* | 1.59 | 1.18 | 2.15 |
| Baseline + DM | *Unexposed* | 1 |  |  |
|  | *Exposed* | 2.15 | 1.68 | 2.76 |
|  | *No DM* | 1 |  |  |
|  | *DM* | 2.31 | 1.76 | 3.03 |
| Baseline + HF | *Unexposed* | 1 |  |  |
|  | *Exposed* | 2 | 1.55 | 2.57 |
|  | *No HF* | 1 |  |  |
|  | *HF* | 3.55 | 2.47 | 5.09 |
| Baseline + HT | *Unexposed* | 1 |  |  |
|  | *Exposed* | 2.56 | 1.99 | 3.29 |
|  | *No HT* | 1 |  |  |
|  | *HT* | 0.43 | 0.34 | 0.54 |
| Baseline + IHD | *Unexposed* | 1 |  |  |
|  | *Exposed* | 2.3 | 1.8 | 2.96 |
|  | *No IHD* | 1 |  |  |
|  | *IHD* | 1.49 | 1.15 | 1.94 |
| Baseline + NSAIDs | *Unexposed* | 1 |  |  |
|  | *Exposed* | 2.22 | 1.73 | 2.84 |
|  | *No NSAIDs* | 1 |  |  |
|  | *NSAIDs* | 0.81 | 0.2 | 3.24 |
| Baseline + Anti-hypertensives | *Unexposed* | 1 |  |  |
|  | *Exposed* | 1.81 | 1.38 | 2.36 |
|  | *1* | 1 |  |  |
|  | *>= 2* | 1.65 | 1.3 | 2.1 |
| Baseline + GP Consultations | *Unexposed* | 1 |  |  |
|  | *Exposed* | 2.23 | 1.74 | 2.85 |
|  | *<10* | 1 |  |  |
|  | *10-19* | 1.5 | 1.11 | 2.04 |
|  | *20-29* | 2.06 | 1.47 | 2.88 |
|  | *>=30* | 2.55 | 1.83 | 3.57 |
| Baseline + SBP | *Unexposed* | 1 |  |  |
|  | *Exposed* | 2.42 | 1.86 | 3.14 |
|  | *<120* | 1 |  |  |
|  | *120-139* | 0.65 | 0.39 | 1.06 |
|  | *140-159* | 0.51 | 0.31 | 0.83 |
|  | *>=160* | 0.54 | 0.33 | 0.88 |
| Baseline + Smoking | *Unexposed* | 1 |  |  |
|  | *Exposed* | 2.21 | 1.72 | 2.83 |
|  | *No* | 1 |  |  |
|  | *Yes* | 1.42 | 1.05 | 1.92 |
|  | *Ex* | 1.4 | 1.09 | 1.79 |
| Basline + GFR | *Unexposed* | 1 |  |  |
|  | *Exposed* | 2.21 | 1.72 | 2.83 |
|  | *>=60* | 1 |  |  |
|  | *45-59* | 1.25 | 0.92 | 1.69 |
|  | *< 45* | 1.96 | 1.14 | 3.39 |
| Full Model | *Unexposed* | 1 |  |  |
|  | *Exposed* | 1.64 | 1.2 | 2.23 |
| Full Model (inc meds*exposure) | *1 (exposed)* | 1.29 | 0.9 | 1.86 |
|  | *>= 2 (exposed)* | 2.31 | 1.15 | 4.65 |
